# The Role of microRNAs in Metabolic Syndrome-Related Oxidative Stress

**DOI:** 10.3390/ijms21186902

**Published:** 2020-09-20

**Authors:** Adam Włodarski, Justyna Strycharz, Adam Wróblewski, Jacek Kasznicki, Józef Drzewoski, Agnieszka Śliwińska

**Affiliations:** 1 Department of Internal Diseases, Diabetology and Clinical Pharmacology, Medical University of Lodz, 92-213 Lodz, Poland; jacek.kasznicki@umed.lodz.pl; 2 Department of Medical Biochemistry, Medical University of Lodz, 92-215 Lodz, Poland; adam.wroblewski@stud.umed.lodz.pl; 3 Central Teaching Hospital of the Medical University of Lodz, 92-213 Lodz, Poland; jozef.drzewoski@umed.lodz.pl; 4 Department of Nucleic Acid Biochemistry, Medical University of Lodz, 92-213 Lodz, Poland

**Keywords:** microRNAs, oxidative stress, reactive oxygen species, prooxidant enzymes, antioxidative enzymes, antioxidative genes, metabolic syndrome, obesity, insulin resistance, diabetes

## Abstract

Oxidative stress (OxS) is the cause and the consequence of metabolic syndrome (MetS), the incidence and economic burden of which is increasing each year. OxS triggers the dysregulation of signaling pathways associated with metabolism and epigenetics, including microRNAs, which are biomarkers of metabolic disorders. In this review, we aimed to summarize the current knowledge regarding the interplay between microRNAs and OxS in MetS and its components. We searched PubMed and Google Scholar to summarize the most relevant studies. Collected data suggested that different sources of OxS (e.g., hyperglycemia, insulin resistance (IR), hyperlipidemia, obesity, proinflammatory cytokines) change the expression of numerous microRNAs in organs involved in the regulation of glucose and lipid metabolism and endothelium. Dysregulated microRNAs either directly or indirectly affect the expression and/or activity of molecules of antioxidative signaling pathways (SIRT1, FOXOs, Keap1/Nrf2) along with effector enzymes (e.g., GPx-1, SOD1/2, HO-1), ROS producers (e.g., NOX4/5), as well as genes of numerous signaling pathways connected with inflammation, insulin sensitivity, and lipid metabolism, thus promoting the progression of metabolic imbalance. MicroRNAs appear to be important epigenetic modifiers in managing the delicate redox balance, mediating either pro- or antioxidant biological impacts. Summarizing, microRNAs may be promising therapeutic targets in ameliorating the repercussions of OxS in MetS.

## 1. Introduction

Metabolic syndrome (MetS) constitutes a cluster of at least three out of five of the conditions including central obesity, high blood pressure, high blood sugar, high serum triglycerides, and low serum high-density lipoprotein (HDL). It is estimated that at least one third of European and American populations and 27% of Chinese population suffer from MetS [1,2]. The most popular definition used for surveys and health care plan is by IDF (International Diabetes Federation) 2006 [3,4]:

Waist >  94 cm (men) or >  80 cm (women) in Europe, > 102 cm (men) or > 88cm (women) in USA, > 90 cm (men) or > 80 cm (women) in Asia, along with the presence of 2 or more of the following:
Blood glucose greater than 5.6 mmol/L (100 mg/dl) or diagnosed diabetes
HDL cholesterol <  1.0 mmol/L (40 mg/dl) in men, <  1.3 mmol/L (50 mg/dl) in women or drug treatment for low HDL-C
Blood triglycerides (TG) >  1.7 mmol/L (150 mg/dl) or drug treatment for elevated triglycerides
Blood pressure >  130/85 mmHg or drug treatment for hypertension (HT)

The incidence of MetS is rising worldwide contributing not only to the increased morbidity and mortality but also to the dramatic increase of treatment costs. Except for pharmacological management, current therapeutic methods include primarily lifestyle changes and diet. Unfortunately, most patients do not follow these recommendations. Bariatric surgery, reserved for patients with morbid obesity, made significant progress in the treatment of obesity and related metabolic disorders, however, it is associated with serious risk and side effects. The molecular mechanisms of obesity and other features of MetS are not yet fully elucidated, while many studies indicate the increasing involvement of epigenetics including microRNAs (miRNAs) [5]. These non-coding single-stranded RNAs, approximately 19–25 nucleotides long, are involved in the transcriptional and post-transcriptional regulation of gene expression by specific interactions with target genes [6,7]. miRNAs play important regulatory roles in a variety of physiological as well as pathological processes including adipocyte differentiation, metabolism, appetite regulation and OxS [8]. OxS is a vital phenomenon occurring in plethora of metabolic disorders including type 2 diabetes (T2DM), obesity and cancer [9,10]. Numerous studies have recently reported a link between OxS and miRNAs, the pivotal regulators of different aspects of glucose homeostasis, such as peripheral insulin signaling, β-cells function, lipogenesis, and chronic inflammation [6,11,12,13,14,15]. For instance, miRNAs directly and indirectly influence he expression level or activity of antioxidative defense genes or ones generating ROS, thereby having an impact on redox balance [16,17,18,19,20,21,22,23]. Moreover, ROS potently affect miRNAs expression levels [24,25,26,27,28]. Recently, 10 circulating miRNAs were indicated to be markers of MetS in adolescents suffering from morbid obesity and miR-122 was reported to predict the risk of development of T2DM and MetS in general population [29,30]. Investigation of miRNAs and their targets may potentially identify new pathways involved in the pathogenesis of metabolic diseases, improving our understanding of the molecular mechanisms influencing the relationship between miRNAs and OxS in MetS. This in turn could be useful for the development of new therapeutic approaches that enable the effective treatment of IR and impaired metabolism of white adipose tissue (WAT) under metabolic stress. The purpose of this review is to present the progress made in this field, describing mechanistic miRNAs-driven gene expression regulation during OxS and MetS progression.

## 2. Overview of Oxidative Stress

OxS, as a prolonged state of a disbalance between the oxidative and antioxidative systems of the cells, results in the overproduction of free radicals and reactive oxygen species (ROS), e.g., superoxide anion (O2^−•^), hydroxyl radical (•OH), singlet oxygen (^1^O_2_), hydrogen peroxide (H2O2), hypochlorous radical (ClO^−^), peroxinitrite radical (ONOO^−^), and nitric oxide (NO)). Nevertheless, O2^−•^ constitutes the precursor for H2O2, •OH, and ONOO^−^ [31]. ROS could attack virtually all types of biological molecules, leading to further cellular and tissue damage. Lipid peroxidation (LPO) is an autocatalytic process generating reactive aldehydes, e.g., malondialdehyde (MDA), trans-4-oxo-2-nonenal (4-ONE) and trans-4-hydroxy-2-nonenal (4-HNE). Protein carbonylation appears due to protein oxidation via ROS (“direct protein carbonylation” of prolines, lysines, threonines, and arginines). The “secondary protein carbonylation” occurs non-oxidatively within side chains of arginines, lysines, and cysteines with adduction of reactive carbonyl species originating from products of LPO as well as autooxidation of carbohydrates (methylglyoxal, glyoxal) [32]. Genotoxic stress is elicited upon oxidative damage to nitrogenous bases within DNA. The most well-known one, 8-oxo-7,8-dihydroguanine (8-oxoG), is perceived as an indicator of whole-body marker of OxS in urine [33].

In general, elevated level of ROS is capable of posing tremendous threat to each cell due to altering its function, metabolism, cell cycle as well as introducing genetic mutations and triggering apoptosis. Importantly, ROS modulate function of cells through modifying proteins at the posttranslational level via phosphorylation and sulfonylation, nitrosylation, carbonylation, and glutathionylation [34,35]. OxS is generated by incomplete reduction of oxygen as electrons flow from one complex to the next in various dynamic intracellular organelles such as the endoplasmic reticulum (ER), lysosomes and mitochondria as by-products of oxidative protein folding, dysfunctional autophagy, mitochondrial respiration and detoxification [10,36]. The essential sources of ROS are the following enzymes: the nicotinamide adenine dinucleotide phosphate (NADPH) oxidase family of enzymes (NOX), as well as cyclooxygenases, xanthine oxidase (XO), myeloperoxidase (MPO), lipooxygenases and nitric oxide synthase (NOS) [37]. Enzymes critical for the strategy of preventing ROS formation are: superoxide dismutases (SODs), glutathione peroxidases (GPx), catalase (CAT) and glutathione reductase (GR) [38]. In Eukaryotes, the most powerful antioxidative enzyme (SOD) is mainly found in mitochondria (SOD2) and cytosol (SOD1). It catalyzes the dismutation (partitioning) of O2^−•^ into O2 and H2O2. GPxs reduce lipid hydroperoxides to their corresponding alcohols as well as mediate breakdown of H2O2 to water. CAT, located mainly in peroxisomes, also mediates decomposition of H2O2 to water and oxygen [39,40]. GR catalyzes the reduction of glutathione disulfide (GSSG) to the sulfhydryl form of glutathione (GSH), which is a critical molecule in resisting OxS, maintaining the reducing environment of the cell and its proper function. Glutathione can act as a scavenger for hydroxyl radicals, singlet oxygen, and various electrophiles. GSH reduces an oxidized form of the GPx enzyme. It also plays a vast role in metabolism and clearance of xenobiotics, acts as a cofactor for certain detoxifying enzymes (e.g., glyoxalases) along with reducing, and thus, regenerating vast antioxidants such as vitamins C and E [41,42].

Secondly, carbonyl groups are indirectly introduced into proteins by non-oxidative covalent adduction of reactive car-bonyl species (RCS) to side chains of the nucleophilic amino acids arginine, lysine, and cysteine. Secondly, carbonyl groups are indirectly introduced into proteins by non-oxidative covalent adduction of reactive car-bonyl species (RCS) to side chains of the nucleophilic amino acids arginine, lysine, and cysteine. In general, ROS are capable of posing tremendous threat to each cell due to altering its function, metabolism, cell cycle as well as introducing genetic mutations and triggering apoptosis. OxS is generated by incomplete reduction of oxygen as electrons flow from one complex to the next in various dynamic intracellular organelles such as the endoplasmic reticulum, lysosomes and mitochondria as by-products of oxidative protein folding, dysfunctional autophagy, mitochondrial respiration and detoxification [10,36]. The essential sources of ROS generated also within the vascular system are the following enzymes: the nicotinamide adenine dinucleotide phosphate (NADPH) oxidase family of enzymes (Nox), as well as cyclooxygenases, xanthine oxidase (XO), myeloperoxidase (MPO), lipooxygenases and nitric oxide synthase (NOS) [37]. The reactive molecules are formed by reduction–oxidation (redox) reactions in response to the activation of several types of oxidases, that catalyze reactions to neutralize free radicals, either directly or via reactions catalyzed by free metals (e.g., Cu, Fe, Cd and Pb) or metals-containing components. These enzymes include: superoxide dismutases (SOD), glutathione peroxidases (GPx), glutathione reductase (GSR), catalase (CAT). SOD is an enzyme that catalyzes the dismutation (or partitioning) of the O2 ^−•^ into ordinary molecular oxygen (O2) and H2O2. O2 ^−•^ is produced as a by-product of oxygen metabolism and, if not regulated, causes many types of cell damage [43]. Three forms of SOD are present in humans, all other mammals, and most chordates. SOD1 is located in the cytoplasm, SOD2 in the mitochondria, and SOD3 is extracellular. GPxs are enzymes with peroxidase activity with 8 isozymes, which are encoded by different genes and the biochemical function of GPx is to reduce lipid hydroperoxides to their corresponding alcohols and to reduce free hydrogen peroxide to water. Mammalian GPx1, GPx2, GPx3, and GPx4 have been shown to be selenium-containing enzymes, whereas GPx6 is a selenoprotein in humans with cysteine-containing homologues in rodents [44]. Glutathione reductase (GSR) catalyzes the reduction of glutathione disulfide (GSSG) to the sulfhydryl form of glutathione (GSH), which is a critical molecule in resisting OxS, maintaining the reducing environment of the cell and its proper function. Glutathione can act as a scavenger for hydroxyl radicals, singlet oxygen, and various electrophiles. Reduced glutathione reduces the oxidized form of the enzyme glutathione peroxidase, which in turn reduces H2O2. In addition, it plays a key role in the metabolism and clearance of xenobiotics, acts as a cofactor in certain detoxifying enzymes, participates in transport, and regenerates antioxidants such as vitamins E and C to their reactive forms [41]. CAT catalyzes the decomposition of H2O2 to water and oxygen, it contains four iron-containing heme groups that allow the enzyme to react with the H2O2 and is usually located in a cellular organelle called the peroxisome, whose major function is the breakdown of very long chain fatty acids through β-oxidation [39,40].

ROS are known to have dual nature — dysfunctional as well as protective [37]. As reviewed by Burthenshaw et al., peroxynitrite (ONOO−), a product of reaction between O2^−•^ with NO, elicits endothelial NOS uncoupling and limits the amount of NO [37,43] Indeed, ONOO− is involved in oxidation of thiols as well as tyrosines nitration—both eliciting further vasculature damage. Another ROS, H2O2, participates in hypertrophy of vascular smooth muscle cells (vSMCs) and activation of metalloproteinases. While at higher concentrations, it inhibits endothelial NOS (eNOS) via proline-rich tyrosine kinase 2 (Pyk2)-dependent tyrosine 657 phosphorylation [44]. However, H2O2 may be also protective and activate eNOS so as to resist pro-atherogenic effects induced by various factors [45]. By inducing oxidative alterations to thiols on cysteine residues, ROS may also elicit changes in signaling of tyrosine and mitogen-activated kinases (both of which being critical for activation of defense mechanisms and antioxidant enzymes) and activation status of transcription factors regulating vast biological processes e.g. expression of proinflammatory cytokines [46,47].

### 2.1. The Interplay between Oxidative Stress and Metabolic Syndrome

**Figure 1:**
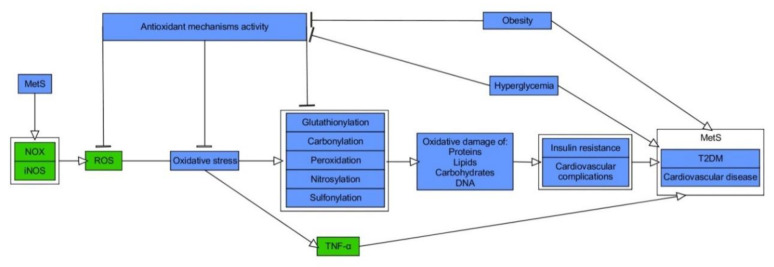
Molecular relationship between oxidative stress and metabolic syndrome. Stimulatory interactions are indicated by arrows and inhibition by T-bars. Actions related to boxes refer to all items inside the box. The figure was made using the PathVisio 3.3.0 free open-source software [64]. MetS–metabolic syndrome, NOX–NADPH oxidase, iNOS–inducible nitric oxide synthase, ROS–reactive oxygen species, TNF-α–tumour necrosis factor-α, T2DM–type 2 diabetes.

While it is broadly accepted that OxS has a role in the pathogenesis of MetS, it is the matter of debate about its causal impact [47]. From the less controversial point of view, OxS constitutes both the consequence and the trigger for MetS, forming a pathogenic vicious cycle initiated in hypertrophic WAT [47,48,49]. Patients diagnosed with MetS exhibit serum hallmarks of redox imbalance in the form of, e.g., increased levels of protein oxidation products, MDA, elevated XO activity, hyperglycemia (HG), elevated TG as well reduced concentrations of HDL-C, vitamin E and C along with declined levels of heat shock response proteins (HSP70) and SOD as compared to healthy probands [50,51,52]. Other studies indicated raised activity of erythrocyte-specific SOD and MPO with elevation of plasma concentrations of H2O2 and MDA in MetS patients in comparison to controls [53,54]. Moreover, a cross-sectional study conducted on the limited number of Japanese MetS patients and healthy subjects indicated an increase of systemic OxS, as determined by urinary 8-epiprostaglandin F2α (8-epi-PGF2α) in single urine samples, being correlated with visceral AT (VAT) accumulation [55]. MetS patients, including those with T2DM, also exhibited elevation of plasma thiobarbituric acid reactive substances (TBARS), protein carbonylation products, and NOx, where the latter indicated the phenomenon of nitrosative stress (NS) [56,57,58]. Additionally, study on MetS and healthy subjects reported raised values for advanced oxidation protein products (AOPP) and pro-oxidant-antioxidant balance (PAB), in plasma and serum, respectively [59]. Importantly, regression analysis indicated positive and independent association between MetS and higher PAB values [56]. Another report showed that levels of ischemia modified albumin (IMA), a protein oxidation marker typical of hypoxia and acidosis, and AOPP were increased with the number of risk factors for MetS, yet it was more significant for AOPP. The latter was also revealed as an independent determinant for occurrence of MetS in studied population of Poles [60]. Interestingly, according to data obtained by Venturini et al., AOPP are more related to components of MetS than markers of LPO [61]. The elevated release of O2−• from the monocytes of MetS patients, plasma levels of ox-LDL, and nitrotyrosine as compared with healthy probands was also found [62]. Appealingly, Yubero-Serrano et al. investigated the relationship between OxS degree and the number of components of MetS in patients. They indicated that activity of SOD and GPx was substantially declined in patients suffering from 2 MetS components than probands with 4/5 MetS components [63]. The general relationship between OxS and MetS is presented in Figure 1.

### 2.2. Hypertrophic, Hypoxic and Inflamed White Adipose Tissue—The Initial Fire for Pathogenic Vicious Cycle of Oxidative Stress in Metabolic Syndrome

The pathogenic mechanisms of MetS are complex, thus remaining to be fully elucidated. WAT is a structure comprised mainly of adipose-derived stem cells (ASCs), preadipocytes, adipocytes and immune cells, which is responsible for fat storage. It deposits an excess of energy in triglycerides or mobilizes fatty acids (FA) according to current metabolic needs. It is perceived as an immunological organ and it releases polypeptides (adipo-/cytokines) as well as metabolites capable of exerting systemic actions, including body weight/energy balance, appetite regulation, glucose homeostasis, insulin signaling, and blood pressure control [65,66]. The first known adipocyte hormone, leptin, whose genetic absence causes massive obesity, suppresses appetite, while other hormones, like adiponectin, have just the opposite effect [67,68]. Adiponectin increases sensitivity of cells to insulin as well as pancreatic β-cells survival and functionality. Furthermore, it exerts cardio- and vasculoprotective impact along with regulating the function of macrophages [69]. Interestingly, Benrick A et al. determined that overexpression of adiponectin has a positive influence on WAT. It decreases the size of adipocytes, increases mitochondrial density, and mediates transcriptional upregulation of factors related to efficient esterification of free fatty acids (FFA) [70]. In overall, due to its insulin-sensitizing, antioxidative, anti-inflammatory, and anti-atherogenic impact, adiponectin protects against the MetS [71].

However, the primary trigger for most of the pathways investigated in MetS is adiposity, especially visceral one, thus stressing the importance of a high caloric intake as a major causative factor [36,72]. Indeed, prolonged and excessive intake of calories, which exceeds white adipocytes’ storage capacity, induces their hypertrophy and hyperplasia, leading to WAT’s hypoxia, along with consequent necrosis and apoptosis of fat cells. These events elicit burst of OxS and M1 type macrophages’ infiltration [73]. Both macrophages and adipocytes produce and secrete proinflammatory adipo-/cytokines and chemokines (e.g., resistin, visfatin, tumour necrosis factor α (TNF-α), monocyte chemoattractant protein-1 (MCP-1, also known as CCL2), interleukin 1β (IL-1β), plasminogen activator inhibitor-1 (PAI-1), interleukin 6 (IL-6), retinol-binding protein 4 (RBP4), and C-reactive protein (CRP)). Hypertrophic WAT is characterized by upregulation of CC chemokines (CCL1/2/3/5,7,8) and their respective receptors (CCR1/2/3/5), the molecules responsible for trafficking leukocytes for the site of inflammation, while CCL2/CCR2 axis is a major one for recruitment of macrophages into WAT [74,75]. Proinflammatory cytokines induce signaling pathways of c-Jun N-terminal kinase (JNK) and IκB kinase-β (IKK-β), while the latter activates a transcription factor involved in production of cytokines and proinflammatory factors, called nuclear factor kappa-light-chain-enhancer of activated B cells (NF-κB) [76,77]. NF-κB is an inducible transcription factor and master regulator of inflammation due to inducing macrophages polarization as well as expression of numerous cytokines (e.g., IL-1, IL-6, TNF-α) and chemokines (e.g., MCP-1, IL-18) [78]. Once secreted, these cytokines activate their extracellular receptors. Simultaneously, the levels of anti-inflammatory adipokines, e.g., adiponectin, omentin decrease. The above biological events trigger a redox imbalance between ROS production and their scavenging, leading to induction of low grade chronic inflammation and intensification of OxS–paving the way for MetS [72,76,79,80]. These phenomena vastly affect WAT, which becomes insulin-resistant, and initiates hyperinsulinemia, enhances lipolysis, increases levels of circulating FFA and their deposition in muscles, liver and pancreas, followed by lipotoxicity, elevated production of glucose due to increased gluconeogenesis and glycogenolysis, and finally, systemic IR and HG [72,81]. Furthermore, atherogenic dyslipidemia, endothelial dysfunction, introduction of hypercoagulable state and HT are observed, thus probably presenting almost the entire spectrum of MetS components [3].

Moreover, the sources of adipose ROS are diversified, being under control of both hormonal and metabolic determinants [82]. Aside from inflammatory cells, mitochondria, as mini factories for ATP production due to oxidative phosphorylation, constitute a major producer of superoxide anions which are converted into H2O2 via SOD2 [83]. It was reported that mitochondrial dysfunction and consequent increased levels of ROS repress insulin signaling as well as production of adiponectin promoting IR in fat cells [84]. Moreover, WAT is the source of ROS-generating enzymes such as NOX, xanthine dehydrogenase/oxidoreductase system (XOR), endoplasmic reticular oxidoreductin 1 (ERO1), pyruvate dehydrogenase (PDH), nicotinamide nucleotide transhydrogenase (NNT) [82]. Interestingly, diet-induced obesity (DIO) in mice supported the elevation of mitochondrial ROS generated by fat cells, thus accelerating mitochondrial uncoupling, biogenesis, and fatty acid oxidation so as to prevent from weight gain and serving as an adaptive mechanism. In other words, the deficiency of SOD2 in adipocytes resulted in increased superoxide levels but simultaneously the lack of IR and increased body mass in spite of obesogenic conditions [85]. Currently, ROS are perceived as second messengers which may facilitate resistance to stress in WAT. However, biological outcomes of short-term and long term-ROS are fully different. For instance, while the first one can be produced upon insulin stimulation, the latter deteriorates insulin response leading to WAT dysfunction [83].

### 2.3. Insulin Resistance, Hyperglycemia and Oxidative Stress

**Figure 2:**
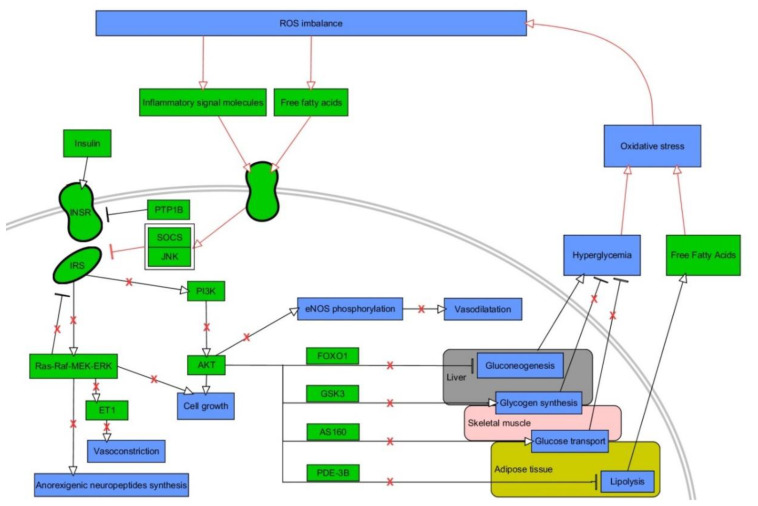
A vicious cycle between oxidative stress and insulin signaling. Stimulatory interactions are indicated by arrows and inhibition by T-bars. Actions related to boxes refer to all items inside the box. Pathological interactions are highlighted in red as well as red crosses denoting withdrawal of physiological insulin signaling. Processes/phenomena are highlighted in blue and proteins/compounds are highlighted in green. The figure was made using the PathVisio 3.3.0 free open-source software [64]. INSR—insulin receptor, IRS—insulin receptor substrate, PTP1B—protein tyrosine phosphatase 1B, SOCS—suppressor of cytokine signaling, JNK–c-Jun N-terminal kinase, PI3K—phosphoinositide 3-kinase, AKT—protein kinase B, FOXO1—forkhead box protein O1, GSK3—glycogen synthase kinase 3, AS160—Akt substrate of 160 kDa, PDE-3B—phosphodiesterase 3 B, eNOS—endothelial nitric oxide synthase.

IR is an impaired response of the body to insulin, resulting in elevated levels of glucose in the blood (a key component of T2DM and MetS) [86]. OxS has been recognized as especially important mechanism in IR [87]. The hormone insulin features a pivotal role in maintaining physiological levels of blood glucose through various effects on insulin target cells [88]. For instance, it elicits vasodilatory as well as vasoconstrictive effects due to the stimulation of endothelial cells for the release of endothelin and nitric oxide, thus increasing the distribution of glucose from blood to organs [89]. Insulin is also critical for highly insulin-sensitive cells, such as muscle, hepatic, and fat ones. Transduction of insulin signal takes place via transmembrane insulin receptors (INSR), whose activation involves dimerization and autophosphorylation of tyrosines located on the intracellular part of receptors due to their kinase activity [88]. Phosphorylated tyrosines are used by adaptor proteins, such as widely known insulin receptor substrates (IRS), as docking sites. These molecules also undergo phosphorylation and mediate the signal via two major pathways: phosphatidyl inositol 3-kinase (PI3K) / protein kinase B (AKT), which activation results in plethora of metabolism-oriented actions, and mitogen-activated kinases (MAPK), which are mainly responsible for growth and differentiation of cells [88]. PI3K conducts phosphatidyl inositol 4,5-biphosphate (PIP2) to phosphatidyl inositol 3,4,5-triphosphate (PIP3) conversion. This is indispensable for plasma membrane recruitment of AKT, followed by phosphorylation of its two specific serine sites by 3-phosphoinositide-dependent kinase-1 (PDK) and mammalian target of rapamycin complex 2 (mTORC 2) [88]. Activated AKT is capable of phosphorylating numerous downstream proteins so as to exert metabolic functions of insulin such as induction of glycogenesis (glycogen synthase (GS)), repression of gluconeogenesis (forkhead box O1 (FOXO1)) or lipolysis (phosphodieterase-3B (PDE-3B)) [77]. Moreover, it initiates intracellular glucose transport due to phosphorylation of Akt substrate of 160 kDa (A160), which is responsible for translocation of glucose transporter 4 (GLUT-4) to cellular membrane in fat and muscle cells [90,91]. IR always involves disturbances in intracellular insulin signaling [92]. It commonly concerns decreased activity or expression of molecules involved in signal transduction (INSR, IRS-1, GLUT-4), decreased expression / translocation of GLUT-4, or increased expression/activity of antagonists of PI3K/AKT pathway, e.g., phosphatase and tensin homolog (PTEN), and polypyrimidine tract binding protein-1 (PTP1B) [93,94,95,96]. The impaired signaling of insulin demands increased concentrations of insulin (hyperinsulinemia). However, an extensive work of pancreatic β-cells to secrete insulin into bloodstream is ultimately pointless, as the vicious cycle of IR is starting to develop, finally leading to a further decrease of available insulin-stimulated GLUT-4 in the cellular membrane and hyperglycemia [97]. Connections between OxS and insulin signaling are illustrated in Figure 2.

Lipotoxicity, which is associated with increased plasma level of FFA and intracellular lipid efflux, is vastly involved in muscle and hepatic IR and dysfunction of β-cells [98]. Moreover, FFA stimulate signaling via protein kinase C (PKC) so as to induce NADPH oxidase-mediated OxS and inflammatory signaling via IKK-β and JNK pathways, which perform direct phosphorylation of IRS [99]. The most well-known indicators of lipotoxicity, intracellular lipid intermediates such as diacylglicerol (DAG) and ceramides, act via either different forms of PKC or via protein phosphatase 2A (PPA2) so as to sequester AKT2 or directly affect IRS proteins [98,100]. Accumulating amount of data suggests that ROS impair insulin synthesis and secretion and induce IR [31,101]. Furthermore, in the course of IR, hyperinsulinemia makes PI3K phosphorylate Rac Family Small GTPase 1 (Rac1) protein instead of PIP2, thus potentiating activity of NOX4 and elevating ROS production [102]. OxS impairs intracellular signaling of insulin due to potentiating activity of SH2-containing tyrosine-protein phosphatase (SHO2), PTP1B and glycogen synthase kinase-3 (GSK-3β) [103,104]. One of the major products of LPO, 4-HNE, potentiates activity of GSK-3β, affects IRS, decreases secretion of adiponectin, and induces lipolysis, protein carbonylation and, finally, IR in muscles [83,105]. Reactive aldehydes make adducts with numerous cellular proteins in various cellular compartments. They mediate protein carbonylation which results in either accumulation or accelerated degradation of affected proteins, enzymes inactivation, changes in gene expression and mitochondrial dysfunction [32]. For instance, several proteins associated with insulin signaling, lipotoxicity, and response to cellular stresses were reported to be carbonylated in WAT of obese insulin-resistant mice, while fatty acid-binding protein was proved to be carbonylated by 4-HNE in vivo [106]. Indeed, protein carbonylation is becoming a more and more studied component of IR and T2DM pathogenesis [32]. Finally, it is worth highlighting that IR is connected with the coordinated interaction among oxidative, nitrosative, genotoxic, carbonyl, and ER stress [86].

HG is a trigger for generation of ROS at the amount which could not be managed by antioxidative system [104]. The central role for HG-mediated OxS is thought to be dependent on the inhibition of glyceraldehyde-3 phosphate dehydrogenase (GAPDH) and accumulation of GAP that upregulates some pathways branching of glycolysis. Namely, HG enhances production of ROS via increasing flux into the polyol, hexosamine and the glyceraldehyde autoxidation pathways as well as activating DAG/PKC signaling pathway, and stimulating formation of advanced glycation end (AGE) products [83,104]. Thus, HG is capable of activating numerous pathways associated with inflammation and OxS, e.g., an activation of PKC stimulates NOX enzymes and lipoxygenases [107]. Excessively generated sorbitol (polyol pathway) mediates activation of p38 MAPK and JNK – core proteins in inflammatory response [108]. The formation of glyoxal (product of glucose autooxidation) and methylglyoxal (product of GAP dephosphorylation) take part in the formation of AGE products. Both precursors bind to specific AGE receptors (RAGE) or interact with various biomolecules thus accelerating OxS via PKC-dependent or independent pathways [104]. Interestingly, methylglyoxal itself affects interaction of insulin with its receptor [109]. AGE/RAGE pathway promotes vascular endothelium’s expression of MCP-1, known to indicate vascular endothelial dysfunction and prothrombotic impact [110]. Furthermore, it is also involved in promoting expression of NF-κB via toll-like receptor 4 (TLR4) pathway [87]. It is to be noted that the early glycation of proteins can be also reversible, as in Schiff bases or Amadori adducts, including a marker of diabetes, glycated hemoglobin (HbA1C) [111]. For thoroughly depicted net of interactions among ROS, IR, HG, and inflammation, see the review by Luc et al. [112].

### 2.4. Dyslipidemia and Oxidative Stress

Dyslipidemia in MetS is a state with elevated level of plasma TG associated with increased level of very low-density lipoprotein (VLDL), small, dense LDL (sdLDL-C), FFAs and low HDL cholesterol level that promotes the development of atherosclerosis [113]. Indeed, oxidatively modified LDL (ox-LDL) is an important player in inducing the process of atherosclerosis as it affects expression of adhesive molecules, cytokines and growth factors and changes function of important vasoactive molecules such as NO, angiotensin II (Ang II) or endothelin 1 (ET 1) [114,115]. Interestingly, treatment of MetS patients with rosuvastatin causes beneficial effect not only on levels of ox-LDL, HDL, and inflammatory markers, but also ameliorates total antioxidant capability [116]. Furthermore, high cholesterol level is a promoter of OxS in endothelial cells [117]. In an extensive review, Spahis et al. enumerated several types of connection between dyslipidemia and OxS in MetS. For instance, elevation of O2^−•^ by NADPH occurring upon obesity/HT/hypertriglyceridemia, as well as lowered level of bilirubin in MetS (a protective agent against LDL oxidation), magnitude of LDL oxidation being dependent on waist circumference (visceral adiposity) or ox-LDL affecting mitochondrial functionality [47]. Hyperlipidemia triggers elevation of ROS and proinflammatory cytokines which may be causative factors for lipotoxicity, being predominantly known to be caused by increased rate of lipolysis and repressed synthesis of TG in obesity-affected, insulin-resistant WAT, triggering increased levels of circulating FFA and accumulation of lipids in non-adipose organs (e.g., liver, pancreas, muscles) [81,118]. For instance, in the recent study by Feillet-Coudray et al., a high-fat high-fructose diet in Wistar rats led to excessive weight gain along with glucose intolerance and hepatic steatosis with elevation of ceramides and DAG (lipotoxicity indicators). Moreover, there was an increase in hepatic NOX activity and protein level of IL-6 along with decrease of total GSSG and GSH as well as activity of SOD and GPx. As these phenomena were associated with moderately marked OxS and inflammation, metabolic alterations were rather suggested to be the trigger, yet, not the cause of OxS [119]. Interestingly, FFAs were reported to be capable of activating renin-angiotensin system (RAS) in mice adipocytes (3T3L1) by TLR4/NF-κB pathway [120]. In fat cells, RAS is connected with the impairment of preadipocytes differentiation, promotion of lipolysis, along with OxS and inflammation.

### 2.5. Hypertension and Oxidative Stress

There are numerous studies which reported persistently increased ROS levels in HT, along with vast improvement upon antioxidative treatment [121,122,123,124,125,126,127]. HT is connected with vascular remodeling, increased vasoconstriction and arterial stiffness, activation of immune cells, renal dysfunction, and excitation of sympathetic nervous system. Above phenomena are inseparably connected with endothelial dysfunction, LPO, inflammation, fibrosis and more, thus favoring the notion that OxS constitutes a common molecular phenomenon in multifactorial pathogenesis of HT [122]. Cardiovascular cells generate ROS due to action of several major enzymes: NOX, XOR, ERO, and uncoupled NOS. While NOX-mediated ROS production is a prevailing one in HT, more and more pieces of evidence suggest its crosstalk with mitochondrial and ER-specific ROS due to phenomenon of ROS-induced ROS release (RIRR) [122,128,129]. Specifically, in the recent review by Touyz et al., OxS was shown to be connected with HT via several ways [122]. Firstly, prohypertensive factors such as salt, aldosterone, Ang II, and ET-1 activate NOX enzymes and ROS production, being a trigger for mitochondrial and ER-located ROS formation, all of which are interconnected with inflammation and immune activation, and consequently, HT. Here, it is worthwhile to enumerate the increased expression of proinflammatory factors (e.g., TNF-α, IL-1/6), adhesion molecules, and activation of signaling via proinflammatory pathways (e.g., NF-κB, JNK). Secondly, elevation of vascular ROS elicits activation of Ca^2+^ channels and further activation of Ca^2+^-sensitive NOX enzymes. Thirdly, Ang II- and ET-1-dependent signaling via their G-coupled receptors promotes transactivation of growth factor receptors (e.g., insulin-like growth factor 1 (IGF-1R), platelet-derived growth factor receptor (PDGFR)) through various mechanisms, triggering activation of signaling via PI3K/AKT and MAPK pathways [122]. Moreover, under physiological conditions, eNOS produces NO, a molecule of critical importance for vasorelaxation. Under NOX-initiated OxS, eNOS produces superoxide, rather than NO, being called uncoupled eNOS and contributing to a sustained increase of ROS levels [130]. Finally, as thoroughly described by Spahis et al., the accumulation of ox-LDL in vascular endothelium is a source of mitochondria-derived OxS in HT [47].

## 3. Overview of miRNAs—The Focus on Interplay with Oxidative Stress

**Figure 3:**
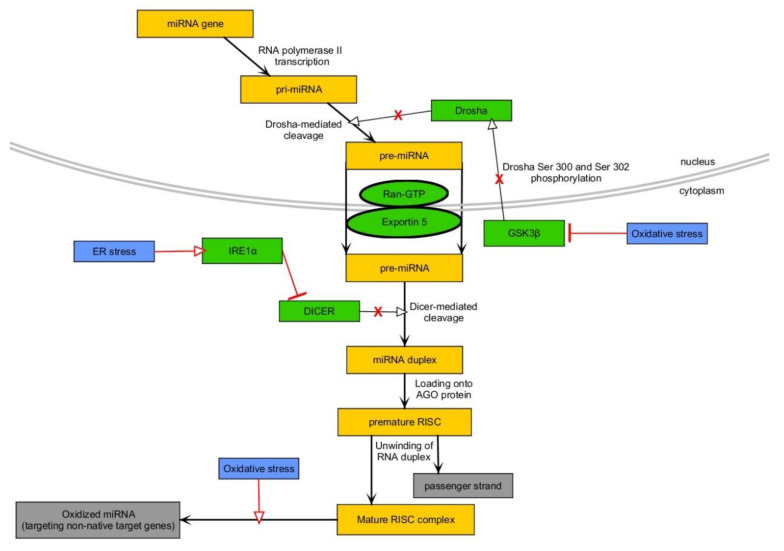
The impact of redox imbalance on canonical pathway of miRNAs biogenesis. For more detailed description see Section 3. Stimulatory interactions are indicated by arrows (except for the sharp black arrows) and inhibition by T-bars. Arrows which are crossed out denote the withdrawal of the stimulation. Red interactions and crosses mark the impact of oxidative stress. Processes/phenomena, elements concerning subsequent stages of produced miRNA, side product of miRNA biogenesis, proteins/compounds are highlighted in blue, yellow, grey and green, respectively. The figure was made using the PathVisio 3.3.0 free open-source software [64]. miRNA—microRNA, pri-miRNA—primary microRNA, pre-miRNA—premature microRNA, Ran-GTP—Ras-related nuclear protein-guanosine-5’-triphosphate, GSK3β—glycogen synthase kinase 3 beta, Drosha—Drosha class 2 Ribonuclease III, DICER—Dicer 1, Ribonuclease III, RISC—RNA-induced silencing complex, IRE1α—inositol-requiring enzyme 1 α.

miRNAs are sophisticated gene expression modifiers which influence expression of majority of protein-coding genes in human [131]. First miRNA molecule was discovered in *Caenorhabditis elegans* and called lin-4. However, miRNAs are found in most of Eukaryotes [132,133]. It is estimated that miRNA-coding genes constitute up to 1% of sequences of human genome [131]. Functional and mature miRNAs are formed in two ways such as canonical and non-canonical, yet, the latter one is typical of approximately 1% of miRNAs [134,135]. The canonical pathway involves RNA polymerase II-based transcription, resulting in the formation of long primary miRNA (pri-miRNA). The latter becomes subjected to 5′capping and polyadenylation before its processing via heterotrimeric assembly (one Drosha molecule and two DiGeorge syndrome chromosome region (DGCR) proteins) called microprocessor [136]. Drosha performs precise endonucleolytic cleavage of pri-miRNA, thus generating precursor miRNA (pre-miRNA) which is transported to cytoplasm via exportin/RanGTP complex. The next step is focused on cleaving both strands of pre-miRNA via Dicer 1, Ribonuclease III (DICER) [137]. This results in formation of double-stranded RNA possessing 3′overhangs and composed of guide and passenger strands. Pre-miRNA, along with DICER, transactivation response element RNA-binding protein (TRBP) and PACT (PKR-Associated Protein X), is loaded into Argonaute (AGO) protein in the process of ATP-dependent premature RNA-induced silencing complex (RISC) formation [137]. Then, unwinding of miRNA duplex is performed in an ATP-independent manner, thus leaving only one strand, typically a guide one [138]. The biogenesis and activity of miRNAs are subjected to tight control via several mechanisms, including the existence of miRNA polymorphisms and epigenetics-based control of miRNA transcription (histone and DNA modifications) or those affecting processing/maturation/activity of miRNAs (e.g., RNA editing) [139]. Subcellular localization of miRNAs is of critical importance—they are able to act and shuttle between nucleus and cytoplasm (mitochondria, rough ER, Golgi network, and more) [7,11]. Hence, it is becoming more and more evident that miRNAs regulate expression of their targets at the transcriptional and post-transcriptional level [7]. Commonly known activity of miRNAs is directed towards targeting 3′ untranslated regions (UTRs) of mRNAs due to seed region and Watson-Crick pairing. However, it was proven that miRNAs can also influence the expression of transcripts using non-seed sequences as well as via pairing within coding and promoter regions as well as 5′UTRs [7,11]. Aside from gene expression silencing via translational repression or mRNA decay, miRNAs trigger mRNAs sequestration in P-bodies – structures which are regarded as key ones in miRNA-induced molecular phenomena [7]. miRNAs also promote upregulation of gene expression via acting as decoys, enhancing translation, or binding directly to promoter regions [140,141,142]. The great repertoire of available mechanisms of miRNAs’ actions along with presence of miRNAs in various cellular compartments highlight the complexity and context-dependent nature of interactions with their target genes [11]. As each miRNA is capable of regulating expression of numerous targets and each target gene is regulated by numerous miRNAs, these molecules are entangled into networks of signaling pathways eliciting plethora of molecular events (apoptosis, necrosis, glucose, and lipid metabolism, growth, differentiation, and more) [143]. These short RNAs act in a tissue- and cellular-specific manner, while some of them are found in biological fluids such as plasma or serum [11]. Thus, pathologically changed tissue may take advantage of miRNAs and affect other distant tissues. Nevertheless, while numerous studies indicate changes in expression levels of miRNAs in diseased states, their usage as clinical biomarkers (response to treatment, diagnosis, prognosis etc.), in spite of great hope and enthusiasm, is still in its infancy [144,145]. miRNAs take part in the maintenance of redox state, thus being important molecules for dampening, via regulating antioxidative defense system, or exacerbating, via influencing ROS-producing systems, OxS [145,146]. Moreover, miRNAs expression is changed by OxS as well as mediates biological impact of OxS in affected tissues [145,146,147]. Mitochondrial dysfunction, as a source of OxS, is also triggered or exacerbated by miRNA-dependent regulation [145]. Further, miRNAs biogenesis is regulated by OxS, while disturbed miRNAs biogenesis is a source of OxS itself, forming a vicious cycle [148]. For instance, OxS inhibits GSK3β, the molecule responsible for conducting serines phosphorylation of Drosha, enabling its translocation to the nucleus, and thus repressing Drosha-mediated cleavage of pri-miRNAs [149]. Moreover, OxS downregulates DICER and affects its activity leading to disturbance of proper pre-miRNA cleavage, and thus miRNAs expression [146]. ER stress promotes the sustained activation of IRE1α that cleaves pre-miRNAs in sites not typical for DICER, which results in the downregulation of some miRNAs [150]. Finally, ROS are capable of oxidizing miRNAs allowing them for targeting novel genes [146,151]. Canonical miRNA biogenesis along with the impact of OxS are depicted in Figure 3. Due to the fact that MetS pathogenesis is strictly associated with OxS (hypoxia, inflammation, HG and more), there are numerous examples of their interplay with miRNAs, as indicated in the following sections.

### 3.1. miRNAs in MetS—A Link with Obesity and Insulin Resistance/Hyperglycemia-Related Oxidative Stress

The involvement of miRNAs is critically demanded for pancreatic organogenesis, including β-cells formation, as reported based on mice model with DICER deletion. Herein, the endocrine defect was attributed to upregulation of hairy enhancer of split 1 (Hes1), the gene in Notch signaling pathway, which elicited downregulation of its target, neurogenin 3, in endocrine progenitor cells [152]. There are also some pieces of evidence proving that miRNAs could regulate insulin secretion. For instance, miR-375, a molecule involved in pancreatic genesis, was shown to target myotrophin (MTPN) as well as pyruvate dehydrogenase kinase 1 (Pdk1), both responsible for insulin secretion. Moreover, miR-375 is widely known to change expression of genes regulating cell growth and proliferation, thus this molecule is a promising target for therapy of diabetes [153]. The suppression of miR-375 also appears to be the way of enhancing chondrocytes for antagonizing OxS in the course of osteoarthritis [154]. Other miRNAs such as miR-29a/c and miR-9 also control insulin release [155,156]. miR-9, an inflammation-associated miRNA, was indicated to participate in glucose-induced insulin secretion (GIIS) while repressing the expression of syntaxin-binding protein 1 (STXBP1) gene. The latter takes part in fusing insulin secretory granules with plasma membrane of β-cells [156]. Another study has indicated that miR-9 was induced by lipopolysaccharide (LPS) in a MyD88- and TLR4/NF-κB-dependent manner [157]. Insulin production is dependent on, e.g., miR-15, a molecule upregulated in the plasma of T2DM patients and correlated with the severity of the disease [158]. Moreover, in vitro and in vivo studies using animal models implicated pancreatic cells-produced miR-15 into utilizing circulation to affect distantly located cells. Specifically, miR-15 was demonstrated to target AKT3 in retinal cells, and thus, induce OxS and cellular injury [158].

Sirtuin 1 (SIRT1), a NAD^+^-dependent deacetylase and important diabetes-related antioxidative gene, is critical in the regulation of cellular senescence, metabolic memory, apoptosis, glucose, and lipid metabolism, and influencing mitochondrial biogenesis, hypoxia, and angiogenesis [159,160]. Chinese researchers have reported that SIRT1 expression is downregulated in insulin-resistant cells, whereas its over-expression improved insulin sensitivity, possibly via the PTP1B inhibition [161]. 5’ AMP-activated protein kinase (AMPK) is a kinase activating SIRT1 via phosphorylating nicotinamide phosphoribosyltransferase (NAMPT) and increasing intracellular NAD^+^ levels [160]. Activated SIRT1 deacetylates and affects the activity of both members of the peroxisome proliferator-activated receptor gamma coactivator 1-alpha/estrogen-related receptor alpha (PGC1α/ERRα) complex and FOXO1/3, which are essential metabolism regulatory transcription factors [162,163]. SIRT1 uses first pathway, PGC1α/ERRα, so as to induce nuclear factor erythroid 2-related factor 2 (Nrf2), yet, it is also capable of direct activation of Nrf2 using numerous mechanisms [164]. The latter negatively impacts on proinflammatory cytokines (e.g., TNF-α, IL-1) and acts as a master regulator of mitochondrial biogenesis and transcription of genes possesing ARE (antioxidant response elements) (e.g., CAT, GPx, heme oxygenenase (HO-1), SOD1/2, GSH, peroxiredoxin 3/5 (PRDX3/5), thioredoxin-interacting protein (TXNIP), NAD(P)H-quinone oxidoreductase 1 (NQO1), glutathione S-transferases (GSTs), glutamate-cysteine ligase modifier subunit (GCLM), cysteine ligase catalytic subunit (GCLC), glucose-6-phosphate dehydrogenase (G6PD)) [164,165,166,167]. Nrf2 is ubiquitinated and directed to degradation by kelch-like ECH-associated protein 1 (Keap1), while Keap1/Nrf2 pathway constitutes the most important cytoprotective pathway responding to ROS [168]. Interestingly, HG was reported to affect SIRT1/NF-κB axis so as to reduce expression of miR-29, a direct regulator of Keap1, eliciting decline of Nrf2 levels [169]. FOXO1 is a transcription factor vastly contributing to insulin sensitivity, being targeted by miR-205-5p [170]. FOXOs regulate expression of antioxidative genes (e.g., SOD1/2, CAT, PRDX3/5), further reflecting antioxidative impact of SIRT1 signaling, yet SIRT1 is also capable of inducing directly the expression of SOD2 [171].

SIRT1 level is declined in WAT via miR-377-dependent direct targeting, leading to inflammation and IR upon obesity [172]. SIRT1 is also directly regulated by miR-34a in a p53-dependent manner in nonalcoholic fatty liver disease (NAFLD) and diabetic endothelial dysfunction [73,173]. Furthermore, miR-34a was found to be upregulated in HFD-induced obese mice and leptin-deficient genetic obese mice, also being significantly elevated in obesity-induced liver steatosis patients [174,175]. It is well characterized that serum visfatin is increased in patients with MetS [176]. Furthermore, Cheleschi et al. indicated that visfatin significantly increased the expression levels of miR-34a and miR-181a via NF-κB pathway in human osteoarthritic chondrocytes. Moreover, visfatin led to the enhancement of expression levels of SOD2, CAT, Nrf2 and the production of O2^−•^ [177]. HOX transcript antisense RNA (HOTAIR), which is implicated in many diseases, was decreased in response to HG in cardiomyocytes and in the hearts of streptozotocin (STZ)-treated mice. Detailed in vitro studies revealed that HOTAIR served as a sponge for miR-34a, where upregulation of the latter was connected with downregulation of SIRT1, decreased signaling via FOXO1, along with the burst of OxS, inflammation and induction of apoptosis [178]. A similar mechanism of action was reported for another long-non-coding RNA (lncRNA), low expression in glucolipotoxicity-treated beta cells (LEGLTBC), in the course of glucolipotoxicity of pancreatic β-cells [179]. Further, in retinal epithelial cells exposed to HG, another downregulated lncRNA, maternally expressed gene 3 (MEG3), was demonstrated reported to affect miR-34/SIRT1 axis [180]. Elevation of miR-34a was also indicated as one associated with OxS in HG-injured cardiomyocytes and regulation of autophagy pathway via targeting B-cell lymphoma 2 (Bcl-2) and SIRT1 [181]. Numerous other miRNAs, including miR-195, miR-217, miR-155, miR-204-5p, and miR-211, were proven to trigger the downexpression of SIRT1 upon diabetes and its complications [160]. Diabetes-triggered mir-23b-3p was shown to induce OxS via decreasing expression of SIRT1 and Nrf2 [182]. Similarly, HG-upregulated miR-221 was proved to target SIRT1, and thus, affect Nrf2 signaling and induce apoptosis of human retinal microvascular endothelial cells [183]. Recent research revealed that miR-221 is protected from sponging via lncRNA growth arrest-specific 5 (GAS5) in diabetic nephropathy and thus leading to SIRT1 decrease [184]. Moreover, SIRT1 was downregulated in diabetic hearts along with increased levels of ROS due to reduction of miR-22, which utilizes direct interaction so as to increase SIRT1 expression [185]. miR-181a was upregulated by HFD in the liver resulting in decreased SIRT1 and impaired insulin sensitivity [186]. On the other hand, SIRT1 induces miR-182, a direct regulator of NOX4, so as to protect from diabetic keratopathy [187]. SIRT1 was also recently found to be regulated by miR-138 in HG-induced OxS-affected retinal cells [188]. Retinal cells affected by HG were also found to express downregulation of lncRNA small nucleolar RNA host gene 7 (SNHG7), leading to elevation of miR-543 and its direct targeting of SIRT1 and decrease of vascular endothelial growth factor (VEGF) [189]. Further, SIRT1 was downregulated by miR-106b in HG-treated pancreatic cells and islets of diabetic mice being accompanied by potentiated ROS production and impaired function of islets [190]. Similarly, HG-mediated elevation of miR-199a-5p was suggested to be a culprit of SIRT1 reduction along with apoptosis and ROS generation in pancreatic β-cells [191]. Far less studied molecule from sirtuins family, SIRT3, positively regulates FOXO1/3, thus resulting in antioxidative response [192,193]. Expression level of SIRT3 was declined due to upregulation of miR-7977 upon hyperinsulinemia in tubular epithelial cells, while contributing to promotion of OxS [194].

miR-27a, which is expressed at high levels in sera of obese individuals with prediabetes and T2DM, is involved in the management of multiple biological and pathogenic processes such as polarization of macrophages, adipogenesis or production of inflammatory factors [195,196]. Recent study suggested that miR-27a is secreted by adipocytes in a form of exosomes upon obesity, and then, taken up by muscles so as to introduce IR via targeting peroxisome proliferator-activated receptor γ (PPAR-γ) in male C57BL/6J mice [196]. Impressively, this miRNA (i) is correlated with fasting glucose in obese and T2DM patients, (ii) stays in positive correlation with BMI in obese children, and (iii) correlates with IR in HFD-fed mice [195,197,198,199]. Another study also confirmed that miR-27a undergoes upregulation in response to HFD in mice as well as upon exposure to TNF-α in adipocytes. Further, miR-27a was indicated to directly change expression of PPAR-γ and further affect the PI3K/AKT/GLUT-4 pathway so as to initiate IR [200].

Another miRNA important for glucose metabolism is miR-592, which directly targets expression of FOXO1. Yuping Song et al. reported reduction of miR-592 in liver of db/db and HFD-fed mice, but no expression change in WAT. Study results indicated that miR-592 prevents obesity-induced HG, IR and hepatosteatosis [201]. Hence, its increased cellular glucose production and triggered upregulation of gluconeogenic enzymes (phosphoenolpyruvate carboxykinase (PEPCK) and glucose-6-phosphatase (G6Pase)), due to overexpression of FOXO1. Expression of miR-592 was also demonstrated to be repressed in mice hepatic cells by palmitate and proinflammatory cytokines (TNF-α and IL-1β), which are known to activate signaling pathways associated with inflammation (NF-κB, JNKs) and ER stress in hepatocytes [202]. ER stress caused by HG can lead to β-cell dysfunction. Rodríguez-Comas et al. indicated that induction of miR-708-5p becomes reduced upon amelioration of ER stress. Moreover, miR-708-5p was reported as a miRNA with most pronouncedly raised expression in pancreatic cells exposed to hypoglycemia in experiment involving numerous miRNAs. Its overexpression elicited effects typically observed upon low-glucose exposure like reduced rate of β-cells proliferation and increased apoptosis along with declined capacity of insulin secretion [203]. miR-708-5p together with its proven target, neuronatin (NNAT), were upregulated and dowregulated, respectively, in islets of ob/ob mice, being characterized by ER stress occurrence in vivo.

miR-483-5p, a miRNA co-expressed with lipid metabolism-associated gene—insulin-like growth factor *2* (IGF2), directly targets suppressor of cytokine signaling-3 (SOCS3) and induces expression of TNF-α in hepatocytes [204]. A study by Gallo et al. suggested that there is a strong link between miR-483-5p and MetS [205]. Moreover, the upregulation of miR-483-5p appeared to be associated with obesity, IR, and dyslipidemia. It was proven that elevated serum level of miR-483-5p may predict the risk of post-operative atrial fibrillation (POAF) in patients who undergo cardiac surgery [206]. Experimental evidence indicates that OxS can contribute to the pathogenesis of POAF [207].

It has been well-characterized that HFD-fed C57BL/6 mice develop skeletal muscle IR [208,209]. Impressively, study concerning the model showed downregulation of miR-194 in skeletal muscle, which was suggested to elicit an increase in, e.g., glucose uptake, glycolysis rate, glucose oxidation capacity, insulin-induced phosphorylation of AKT and GSK3β, and expression of proteins of mitochondrial oxidative phosphorylation complexes. Its reduced expression was also observed in muscles of patients diagnosed with prediabetes and T2DM. In silico analysis suggested connection among miR-194 and T2DM due to prediction of targeting several important genes critical for insulin (e.g., AKT2, FOXO1, GRB10) and AMPK signaling (e.g., ATM, MAPK1) [210]. Another interesting study showed that upregulation of miR-194 in obese mice causes heart failure and mitochondrial dysfunction [211]. It is well known, that obese heart is characterized by reduced rates of oxidative phosphorylation (OXPHOS) and diminished efficiency in ATP synthesis [212]. Nie et al. established a considerable decrease of mitochondrial complex I activity, oxygen consumption and ATP production in cardiac tissues of obese mice, followed by severe heart injury [211]. Finally, Jaeger et al. revealed elevated serum levels of miR-192, miR-194 and miR-215 linked with the presence of both T1DM and T2DM in patients, whereas miR-192 and miR-194 were correlated with the incidence of new-onset T2DM in 6 years of follow-up. The latter miRNAs were also elevated in diabetic Akt2 knockout mice [213]. Another interesting miRNA associated with IR is miR-802. Yang et al. has shown that overexpression of miR-802 induced in HFD-fed mice reduced the activities of SOD, CAT and GPx enzymes. In consequence, it led to OxS, generation of ROS and potentiated production of LPO. Furthermore, miR-802 induced the expression of phosphorylated p38MAPK and JNK, however, the levels of phospho-ERK remained unchanged. In conclusion, by means of miR-802, HFD may cause IR via activating the JNK and p38MAPK pathways and also suppressing antioxidative enzymes to induce hepatic OxS [214]. HG-promoted OxS along with IR in HepG2 cells was mediated by decreased levels of miR-233, thus preventing miR-233-dependent direct regulation of Keap1 and reducing levels of Nrf2, HO-1 and SOD1 [215]. OxS in the liver of diabetic mice was ameliorated upon transplantation of brown AT (BAT) from healthy donor mice, as indicated by upregulation of Nrf2 and downregulation of NOX2 and NOX4, along with improvement of glucose and lipid metabolism. Detailed studies suggested that circulating miR-99a, whose expression raised, might directly repress the expression of NOX4, thus mitigating OxS in the liver [215,216].

MiR-21 is broadly related to metabolic disorders, e.g., CVD, diabetic retinopathy, kidney fibrosis, atherosclerotic plaques, and β-cell apoptosis, and it has been shown to influence Bcl-2 in type 1 diabetes in vivo models [217,218,219,220,221]. miR-21 regulates genes of critical importance for homeostasis of intracellular ROS, SOD2, Nrf2 and Krev/Rap1 interaction trapped-1 (KRIT1) [222]. La Sala et al. observed upregulation of miR-21 along with increase in OxS in primary pooled human umbilical vein endothelial cells (HUVECs) subjected to constant HG and oscillating glucose exposure [222]. Moreover, in vivo studies revealed that miR-21 was downregulated upon IR in 3T3-L1 adipocytes, while its overexpression led to significant enhancement of insulin-triggered glucose uptake [223]. Interestingly, other studies by La Sala et al. confirmed that elevated levels of miR-21 are associated with increased abundance of ROS and reduced SOD2 antioxidant defense. Moreover, the authors suggested that miR-21 might serve as a predictive parameter for the early detection of glucose imbalances in prediabetic patients [224]. There is also a strong link between obesity and miR-21. Previous studies have shown that miR-21 is over-expressed in human obesity, however, recent results are contrary [225]. Ghorbani et al. presented that the level of miR-21 in non-diabetic obese patients was significantly lower than in non-diabetic lean subjects. In addition, obese diabetic probands had lower level of serum miR-21 as compared to lean diabetic subjects [226]. To sum up, miR-21 might be a negative regulatory factor in MetS.

MiR-200c, miR-21, and miR-146a were identified in H2O2-treated cells and were also elevated in animal models associated with higher OxS [227]. The cluster of miR-200 is a notable player in oxidative response in impaired glucose metabolism. The action of miR-200c was proved to be directed towards targeting zinc finger E - box-binding homeobox (ZEB1) [228]. ZEB1 downregulates E-cadherin and induces epithelial to mesenchymal transition (EMT), which is associated with inhibition of apoptosis. Importantly, HG-triggered cellular stress induces miR-200 family so as to reduce expression of ZEB1 and initiate apoptosis [229,230]. The miR-200 family negatively regulates β-cell survival in T2DM and its overexpression in a mice model elicits the death of β-cells [231].

Piperi et al. have reviewed numerous studies with interplay among AGE/RAGE signaling, ROS and miRNA [232,233]. Thus, AGE/RAGE signaling mediates downregulation of expression of miR-16, miR-200a, miR-200b/miR-200c and miR-205. These epigenetic changes lead to elevated OxS, diabetic nephropathy and dysregulation of inflammatory response [234,235,236,237]. Activation of AGE/RAGE signaling also leads to the upregulation of miR-21, miR-192, miR-30 family, miR-214, miR-221, miR-222, and miR-223 [238,239,240,241].

Glucose oscillations (OG), which were introduced to mimic ones experienced by diabetic patients at daily basis, were suggested to be much more dangerous than constant HG for various cells. The reason for this could be associated with potentiated generation of ROS [242]. Generally, OxS induces antioxidant enzymes, such as SOD, CAT, and GPx, while aiming to protect cells [243]. However, it appears that both chronic HG and OG does not trigger expression changes of SOD2 and CAT, while vastly affecting expression of SOD1 in human endothelial cells [242]. Expression of GPx-1 was also increased upon constant HG. However, OG triggered upregulation of miR-185, which prevented expression changes of its direct target, GPx-1, thus reflecting an impaired response of GPx-1 under these conditions. Interestingly, miR-185 appears to be important for cholesterol metabolism as it targets hepatic scavenger receptor class B type I (SR-BI), leading to reduction in HDL-C uptake by hepatocytes [244]. However, there are other examples of miRNAs which are either downregulated (e.g., mir-29b, miR-200a/b) or upregulated (e.g., miR-155, mir-92a, miR-137) upon hyperglycemic/diabetic milieu in endothelial cells and contributing to endothelial inflammation and/or OxS deterioration. Specifically, these miRNAs were found to regulate redox balance via eNOS (miR-155), AMPKα1 (miR-137), *O*-GlcNAc transferase (OGT) (miR-200a/b) or components of Keap/Nrf2/HO-1 pathway (miR-92a, miR-200a) [245,246,247,248,249,250]. Interestingly, OxS was augmented due to lack of miR-29b-dependent regulation of AKT/eNOS signal pathway, as miR-29b was sponged by lncRNA H19 in vascular endothelium [245]. miR-29b was also found to directly regulate vascular endothelial growth factor A (VEGFA) [245]. Vascular smooth muscle cells are also affected by HG-induced OxS due to reduction of miR-24 and simultaneous upregulation of its direct target, OGT, along with increase of Keap1 and downregulation of Nrf2 and HO-1 [251].

**Figure 4:**
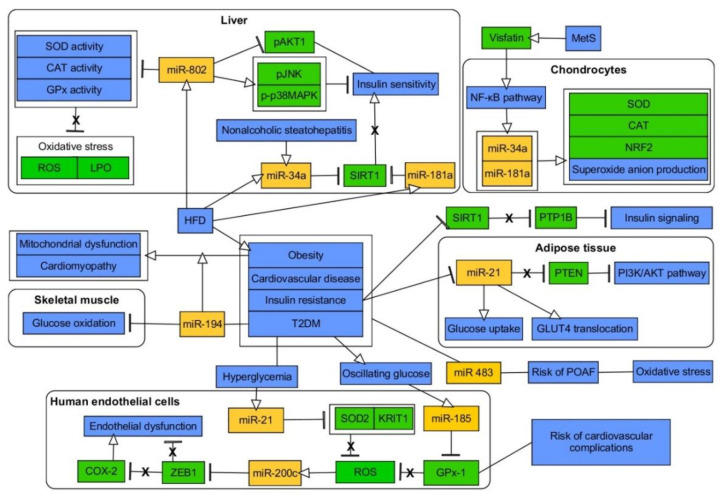
Epigenetic interplay between OxS and MetS. Stimulatory interactions are indicated by arrows and inhibition by T-bars. Actions related to boxes refer to all items inside the box. Arrows and T-bars which are crossed out denote the withdrawal of the respective type of regulation. Processes/phenomena are highlighted in blue, proteins/compounds are highlighted in green and miRNAs are highlighted in yellow. The figure was made using the PathVisio 3.3.0 free open-source software [64]. SOD—superoxide dismutase, CAT—catalase, GPx—glutathione peroxidases, ROS—reactive oxygen species, LPO—lipid peroxidation, pAKT1—phosphorylated protein kinase B, p-JNK—phosphorylated c-Jun N-terminal kinase, p-p38MAPK—phosphorylated p38 mitogen activated kinase, miR—microRNA, SIRT1—sirtuin 1, MetS—metabolic syndrome, NF-κB—nuclear factor kappa-light-chain-enhancer of activated B cells, NRF2—nuclear factor erythroid 2-related factor 2, PTP1B—polypyrimidine tract binding protein-1, PTEN—phosphatase and tensin homolog, PI3K—phosphatidyl inositol 3-kinase, T2DM—type 2 diabetes, HFD—high-fat diet, POAF—post-operative atrial fibrillation, KRIT1—Krev/Rap1 interaction trapped-1, COX-2—cyclooxygenase 2, ZEB1—zinc finger E-box-binding homeobox.

Hyperglycemia is a trigger for OxS and changes in expression of miRNAs in other diabetic complications as well. miR-106a was revealed as downexpressed in the pathogenesis of diabetic peripheral neuropathy, thus contributing to 12/15-lipoxygenase (12/15-LOX)-dependent OxS and NS [252]. miR-590-3p was downregulated in HG-affected retinal microvascular endothelial cells (HRMECs) allowing for increased expression of its target, and activation of NOX4 [253]. HG treatment in retinal endothelial cells evoked reduction of miR-145 together with increase of signaling via TLR4/NF-κB pathway, potentiation of OxS, inflammation and apoptosis. Indeed, miR-145 was proved to directly target TLR4 [254]. Considering retinal pigment epithelial (RPE) cells, miR-455-5p ameliorated OxS and inflammation triggered by HG, as shown by the increased activity of antioxidant defense genes (SOD, CAT, GPx), reduced ROS production, expression of NOX4 and proinflammatory cytokines. This beneficial impact was proven to be mediated by the direct regulation of previously mentioned SOCS3 [255]. In another study, quercetin elicited the upregulation of miR-29b in HG-exposed RPE cells, thus leading to amelioration of ROS production due to miR-29b-dependent beneficial regulation of PTEN/AKT and NF-κ pathways [256]. HG-mediated apoptosis of RPE cells was reported to be, at least partially, accomplished via induction of miR-383, followed by reduction of expression of its direct target, PRDX3, an antioxidant enzyme [257]. A recent study by Jadeja et al. showed that RPE cells-specific miR-144-3p and -5p are subjected to upregulation upon prooxidant stimulation (one typically occurring in diabetic retinopathy or age-related degeneration), being connected with declined levels of Nrf2 and antioxidant genes (GR, GCLC, NQO1) [258]. Both miRNAs were shown to target 3′UTR of Nrf2, yet, only the impact of miR-144-3p was studied using antagomir-based approach and subretinal injection in mice retinas. As expected, downregulation of miR-144-3p exerted an antioxidative impact along with decreased apoptosis and increased retinal function and integrity [258]. Nrf2 is also targeted by miR-93 upon HG in retinal cells due to downregulation of lncRNA MEG3, a direct regulator of miR-93 [259]. Injury and ROS generation induced by HG were also associated with downexpression of miR-26a and possibly through enhancement of signaling via ERK Wnt/β-catenin pathways [260]. Another miRNA, miR-195, was upregulated in the course of diabetic retinopathy leading to aggravated OxS, mitochondrial damage and apoptosis of RPE cells via Bcl-2 targeting [261]. miR-195 was also indicated to be increased in retinas of diabetic mice while directly targeting 3′UTR of mitofusin-2 (MFN-2) [262]. Finally, HG elicited downregulation of miR-130a-3p, and therefore, potentiated signaling via TNF-α/SOD1 pathway leading to excessive ROS and pyroptotic death of RPE cells [263]. The same HG-induced mechanism was shown for podocytes, yet with proved direct impact of miR-130a-3p and miR-301a-3p on TNF-α [264]. Podocyte injury upon HG was also found to be associated with reduced expression of miR-15b-5p. The lack of repression of its direct target, semaphoring 3A (Sema3A), was a culprit for increased ROS production and inflammation in podocytes [265]. Further, patients with diabetic nephropathy were characterized by decline in miR-423-5p, and detailed studies elucidated that HG-elicited injury of podocytes was also attributed to upregulation of direct target of miR-423-5p, NOX4 [266]. Similarly, the upregulation of NOX4 and reduction of its direct regulator, miR-25, was reported in kidneys of diabetic rat model, HG-treated mesangial cells and upon diabetic peripheral neuropathy [267,268]. Expression of another NOX, NOX5, was proved to be rescued by HG-reduced miR-485, its direct regulator, in human mesangial cells, thus enhancing their proliferation and inflammation [269]. NOX4 was also found to be targeted by miR-146a in diabetic nephropathy, while overexpression of miR-146a was accompanied by amelioration of both inflammation and OxS [270,271]. miR-146a is also involved in endothelial inflammation via, among other, regulating NOX4 [272,273]. Diabetic nephropathy-related OxS is also improved upon elevation of miR-214 via using uncoupling protein 2 (UCP2) so as to affect signaling via ROS/AKT/mTOR pathway [274]. miR-140-5p also ameliorated HG-promoted ROS generation, inflammation, and apoptosis in human renal tubular epithelial cells (HK-2) via repressing signaling via TLR4/NF-κB [275]. ROS production along with apoptosis of HK-2 became potentiated upon HG-triggered upregulation of miR-125b, which directly targets a protective molecule, angiotensin-converting enzyme 2 (ACE2) [276]. Finally, further studies on HK-2 cells allowed for finding an lncRNA-miRNA HG-evoked regulatory mechanism of OxS deterioration. Namely, lncRNA GAS5 was decreased upon HG, thus triggering upregulation of miR-452-5p along with inducing pyroptosis, ROS production (measured by level of ROS, MDA and SOD), and inflammation [277]. Another downregulated lncRNA, LINC01619, failed to sponge miR-27a, thus eliciting targeting of FOXO1 and ER stress-induced podocytes injury upon HG [278]. Considering diabetic cardiomyopathy, HG-associated myocardial OxS, fibrosis, hypertrophy and apoptosis were also improved via elevation of miR-203 along with reduced signaling via PI3K/AKT pathway due to reduction of expression of phosphatidylinositol-4, 5-bisphosphate 3-kinase, catalytic subunit alpha (PI3KCA), a direct target of miR-203 [279]. Diabetic hearts were also found to exhibit 14 miRNAs with reduced expression, while 2 of them, miR-92a-2-5p and let-7b-5p, were proved to positively regulate mitochondrial gene cytochrome-b (mt-Cytb). Therefore, reduction of both of these miRNAs elicited excessive mitochondrial ROS production [280]. The contribution of miRNAs in molecular linkage between MetS and OxS is summarized in Table 1 and Figure 4.

### 3.2. MicroRNA in MetS—A Link with Chronic Inflammation Related to Oxidative Stress

As mentioned above, in pathophysiology of MetS a pivotal role besides IR is played by chronic inflammation, hypoxia of WAT and lipid disorders. Herein, miRNAs are known to participate in body weight regulation as well as inflammation. Sheikhansari et al. have shown increased OxS and inflammatory factors in MetS women as compared to healthy probands. Moreover, MetS elicited decreased expression of miR-223 and miR-146a and upregulation of miR-21 in peripheral blood mononuclear cells (PBMCs) [286]. Other studies indicated that downregulation of miR-223 promotes increase of IL-1β and IL-6 production [287]. miR-146a mediates the repression of signaling via IRAK1-TRAF6-NF-κB pathway, and thus its reduced expression augments the inflammatory responses [288].

There are some studies, that provide evidence of the underlying mechanism of miRNAs as prognostic markers of obesity-regulated inflammation process. Marques-Rocha et al. demonstrated that eight weeks of nutritional (Mediterranean-based) intervention was capable of decreasing the white blood cells (WBCs)-specific expression of miR-155-3p and increasing the expression of let-7b in patients with MetS. It was hypothesized that the increased expression of let-7b may contribute to the decrease of ROS production and LPO as MDA and PAI-1 levels decreased after intervention [289]. Previous studies have identified let-7 as a tumor suppressor, being associated with a variety of human diseases, including lung disease, liver fibrosis, CVD, and cancers, and regulating multiple aspects of glucose metabolism [290,291]. Overexpression of miR-155 led to targeting and reduction of CCAAT/enhancer-binding protein β (C/EBP-β) mRNA, which was implicated in the regulation of proinflammatory cytokines during macrophage activation and the acute phase response [292]. Another dietary intervention took advantage of Mediterranean diet phytochemical, hydroxytyrosol (2-[3,4-dihydroxyphenil]-ethanol, one with strong radical scavenging activity [293]. In adipocytes treated with TNF-α as well as in their exosomes, treatment with hydroxytyrosol prevented activation of NF-κB, downregulation of let-7c-5p and upregulation of miR-155-5p and mir-34a-5p along with improvement of ROS production. As suggested by authors, hydroxytyrosol may potentially exert a beneficial paracrine and endocrine impact on distant cells and organs via miRNAs due to similarity of expression changes observed between adipocytes and exosomes [293]. Next interesting dietary intervention revealed that the fruit juice of Actinidia chinensis planch (kiwi fruit) has antioxidant and anti-inflammatory properties in patients with T2DM. After 9 months of intervention, increased expression of miR-424, Keap1, and Nrf2 along with reduced levels of IL-1 β and IL-6 were reported in T2DM patients [294]. Likewise, there is an evidence that high consumption of dietary fructose is a major contributor to MetS. High fructose-induced ROS production, especially superoxide anion, participates in podocyte injury in rodents, thus leading to nephropathy in MetS patients [295]. It is well known that XO is a major source of ROS production under high-fructose condition [296]. Moreover, excessive fructose consumption is an important inductor of WAT accumulation, mediates leptin resistance, increases lipogenesis, hypertriglyceridemia, and increased visceral fat inflammation [297,298,299]. As mentioned earlier, miR-377 evokes WAT inflammation via targeting SIRT1, serving also as a proved regulator of SOD1 and SOD2 [172,300]. Notably, high-fructose treatment increases miR-377 expression in MetS-related injury of glomerular podocytes in vitro and in vivo, also indicating targeting of SOD1 and SOD2. Overexpressed miR-377 triggers activation of O2(-) / p38MAPK / TXNIP / NLR family pyrin domain containing 3 (NLRP3) inflammasome pathway inducing OxS and podocyte injury [301]. Interestingly, Hernández-Díazcouder and collaborators reviewed some of the miRNAs that can be associated with the induction of adipogenesis by high-fructose diet (miR-206, miR-122, miR-33, miR-378a, miR-21, and miR-223) [302]. It has been indicated that DICER and miRNAs can modulate the inflammatory response and lipid metabolism in macrophages to suppress atherosclerosis. DICER, endoribonuclease, which takes part in miRNA biogenesis, stimulates oxidative phosphorylation and mitochondrial fatty oxidation by inducing miR-10. Its targets, two nuclear receptor co-repressors, ligand-dependent corepressor (LCoR) or nuclear receptor corepressor 2 (Ncor2), were shown to attenuate PPAR-γ/(retinoid X receptor (RXR)/PGC1 signaling. Thus, triggering DICER induces mitochondrial oxidative metabolism, leading to decrease of lipid deposition of macrophages. Such metabolic reprogramming also has a protective effect against the macrophage apoptosis. All of mentioned effects of DICER along with reduction of inflammatory response, foam cell accumulation and necrotic core formation restrain atherosclerosis [303].

NAFLD is often a hepatic manifestation of the MetS, however, it can be considered as both a cause and a consequence [304]. The association between NAFLD and MetS components, especially IR, obesity, dyslipidemia, and HT, is more troublesome than previously thought. During the course of NAFLD, DAG elicits activation of PKC-ε, thus, reducing activation of insulin receptor and glycogen synthesis. Moreover, lipogenesis and gluconeogenesis become promoted [92,305]. miR-421 was suggested to mediate OxS occurrence in NAFLD tissues, based on mice model, by directly targeting SIRT3, thus affecting FOXO3 signaling and leading to decrease of CAT and SOD2 [306]. Another miRNA potentiating OxS is HFD-induced miR-34a utilizing direct targeting of NAD+-dependent SIRT1 or indirectly, via reducing NAD+ levels by targeting Nicotinamide phosphoribosyltransferase (NAMPT) [307,308,309]. Downregulation of SIRT1 is connected with repression of fatty acid oxidation via AMPK/ liver kinase B1 (LKB1) and PGC1α/PPARα pathways decreasing malonyl-CoA decarboxylase (MLYCD) and carnitine palmitoyltransferase 1 (CPT1), yet, promotion of lipid synthesis, and thus, inflammation [307]. The connection between lipid accumulation and SIRT1 was also found in studies reporting about the role of miR-23b-3p and miR-9-3p in HepG2 cells [310,311]. Hepatic lipid accumulation is also deteriorated with stimulation of signaling via miR-34/SIRT1/sterol regulatory element-binding protein 1 (SREBP-1) lipogenic pathway in response to high fructose diet [312]. High fructose diet also evoked reduction of hepatic miR-200a and upregulation of its direct target, Keap1. This triggered inactivation of Nrf2 signaling and consequent reduction of HO-1, GST and NQO1 protein levels [313]. Moreover, overexpression of miR-29a was recently suggested to ameliorate HFD-induced weight gain and NAFLD via targeting cluster of differentiation 36 (CD36). Authors suggested that repression of CD36 reduces FA flux into the liver and precludes PPARγ-mediated increase of mtDNA and mitochondrial ROS, being a trigger for inflammatory response [314]. For an extensive review on miR-29a in NAFLD see recent paper by Lin et al. [315]. Further, Albracht-Schulte and collaborators indicated that eicosapentaenoic acid (EPA) targets hepatic miRNA involved in NAFLD pathways in order to improve metabolism and reduce inflammation. Namely, in spite of HFD, EPA treatment led to beneficial reduction of miR-21 being in agreement with data suggesting that mice with mir-21 knockout exhibit diminished hepatic steatosis, inflammation, and lipogenesis [316]. Interestingly, Zhang et al. indicated that miR-101 targets ATP-binding membrane cassette transporter A1 (ABCA1), and therefore may have significant impact on the development of NAFLD and vascular atherosclerosis [317]. ABCA1 protein contributes to lipid removal from the cell and also triggers signaling to enhance the forementioned lipid efflux and induce, e.g., anti-inflammatory effects as well [318]. Additionally, IL-6 and TNF-α treatment lead to upregulation of miR-101. Thus, miR-101 supports intracellular retention of cholesterol upon inflammatory conditions by declining expression level of ABCA1 [318]. The latter is also negatively regulated by miR-758, mir-144, miR-128-2, and miR-145 [125,319,320,321]. Aside from ABCA1, another ABC transporter, ATP-binding cassette sub-family G member 1 (ABCG1), RXRα, and SIRT1 were validated as targets of miR-128-2 in the regulation of cholesterol metabolism [125]. Moreover, D’Amore and collaborators reported that ABCA1 was reduced by miR-9-5p, the target of NF-κB. ABCA1 mRNA levels in CD14+ cells of patients were also negatively correlated to BMI, HOMA-IR, and cardiovascular risk, whereas they were positively correlated to HDL cholesterol and cholesterol efflux. Being overexpressed by CD14+ cells of MetS patients, miR-9-5p could possibly modulate low-grade chronic inflammation response [322].

Another miRNA reported to be involved in MetS and related to OxS is miR-33a-5p. After exposure to inflammatory cytokines (IL-6 or TNF-α) the expression of miR-33a-5p and SREBP-2 was upregulated. Moreover, inflammatory cytokines downregulated expression of ABCA1 and ABCG1 in THP-1 macrophages. Therefore, inflammatory stress caused lipid accumulation possibly by acting on miR-33a-5p and ABCA1 and ABCG1-mediated cholesterol efflux [323]. As mentioned above, stimulation with proinflammatory cytokines is also a trigger for miRNAs dysregulation (e.g., miR-199a, miR-155, miR-146b, miR-130) in white AT and adipocytes, including visceral ones, where miR-155 and miR-130 directly target vast regulator of lipid metabolism, PPAR-γ [293,324,325,326,327]. miRNAs included in this section are briefly summarized in Table 2.

### 3.3. MicroRNA in MetS–A Link with Dyslipidemia/Hypoxia of White Adipose Tissue Related to OxS

Several reports showed the impact of hypercholesterolemia on expression of miRNAs in hearts [21,328,329]. Firstly, cholesterol-enriched diet triggered reduction of miR-25 expression along with upregulation of its target, NOX4, in hearts of male Wistar rats [21]. This led to increase of myocardial OxS/NS as well as diastolic dysfunction. The same research team also evaluated global miRNA expression pattern via microarrays in hypercholesterolemic hearts, and found 47 upregulated (e.g., miR-133b, miR-101a, miR-29b, miR-223, miR-21) and 10 downregulated (e.g., miR-93, miR-25) molecules [329]. Further, hypercholesterolemia suppressed protective upregulation of miR-125b-1-3 upon ischemic preconditioning (IPre) in heart of Wistar rats [328]. Moreover, hypercholesterolemic patients showed reduced expression of miR-98 in both serum and liver along with elevation of miR-98 direct target, SREBP-2. Animals with an overexpression of miR-98 showed declined serum levels of cholesterol, and reduced expression of SREBP-2 together with its two downstream proteins, hydroxy-3-methylglutaryl-CoA reductase (HMGCR) and LDL receptor (LDLR) [330]. High cholesterol diet also triggered elevation of miR-92a in the trunks of zebrafish in the OxS-dependent way [331]. Detailed studies pointed out that OxS promotes SREBP-2-mediated induction of miR-92a expression in endothelial cells. miR-92a targets genes critical for endothelial homeostasis (e.g., SIRT1) and promotes eNOS inhibition along with activation of inflammasome, thus deteriorating endothelial dysfunction upon OxS [331]. Serum expression of another molecule, miR-379, was indicated as a potential NAFLD biomarker, staying in positive correlation with high cholesterol levels, yet, being weakened upon statins treatment [332]. In silico analysis predicted numerous target genes of miR-379 associated with metabolism (e.g., INSR, IGF-1, IGFR, SOCS1, FOXO1) and inflammation including, among others, CAT [332]. Human hepatic cells were reported to express miR-140-5p, which was proved to target LDLR and inhibit uptake of LDL-c. Treatment with simvastatin elicited decline of miR-140-5p, what paves the way for using it as therapeutic strategy to combat hypercholesterolemia along with atherosclerosis [333]. Other miRNAs implicated in potential therapy of hypercholesterolemia are miR-27a and miR-30c. First of them was injected via tail vein into Apoe-/- mice subjected to high cholesterol diet and suggested to diminish plasma lipid levels along with hepatic levels of HMGCR [334]. Further, miR-27a was proved to directly target HMGCR via translational repression and mRNA decay. Moreover, it was observed that hypoxia, a sign of liver damage, induced binding of early growth response protein 1 (Egr1) to promoter regions of miR-27a, leading to its induction, to mediate the downregulation of HMGCR. Finally, increase of miR-27a and decrease of HMGCR were found in livers of three mice models of MetS [334]. Further, C57BL/6J mice with diet-induced hypercholesterolemia showed also amelioration of hypercholesterolemia, yet upon delivery of miR-30c mimic to the liver due to decrease of hepatic lipoprotein production but not LDL clearance [335]. Moreover, pathways enrichment analysis showed that miR-30c suppressed lipid metabolic pathways [335]. miR-30c was also suggested to participate in improvement of atherosclerosis and hyperlipidemia via decreasing lipid biosynthesis and secretion of lipoproteins by downregulating lysophosphatidylglycerol acyltransferase 1 (LPGAT1) and microsomal triglyceride transfer protein (MTP) [336]. Interestingly, hyperlipidemia promoted upregulation of miR-155-5p, serving as an adaptive mechanism of pancreatic β-cells to obesity-triggered IR [337]. This effect was accomplished by targeting v-maf musculoaponeurotic fibrosarcoma oncogene family, protein B (Mafb), which reduces expression of IL-6 leading to inhibition of intra-islet production of GLP-1 [337].

Another interesting report showed, that miR-24 repressed HDL uptake and steroidogenesis possibly by SR-BI [338]. Aside from mediating scrupulous uptake of HDL, SR-BI participates in cell inflammatory responses, e.g., promoting anti-inflammatory response and phagocytes’ survival by efferocytosis of apoptotic cells [339,340]. Wang et al. also reported that upregulation of miR-24 led to decline in expression of lipogenesis-related genes, whereas the expression of genes relevant for cholesterol synthesis increased [338]. Additionally, SR-BI acts as a multiligand membrane receptor. It takes part in recognition, binding as well as uptake of both exo- and endogenous ligands while instigating anti- or proinflammatory response [341]. In consistence with previous miR-24 regulatory effect, Zhigang et al. reported inhibition of SR-BI induced by miR-125a and miR-455 that dramatically repressed lipoprotein-supported steroidogenesis in mouse MLTC cells (a Leydig cell line in which mRNA levels of SR-BI are induced by severalfold in response to cAMP treatment) [342]. Hepatic expression of miR-125a was recently revealed to be declined in models of mice obesity, while its overexpression promoted increase of insulin sensitivity and reduction of hepatic steatosis due to downregulating ELOVL fatty acid elongase 6 (Elovl6) [343]. Interestingly, in silico analysis predicted miR-125a to target other relevant genes for lipid metabolism as well as gluconeogenesis (PEPCK, G6Pase1) [343]. Moreover, in abdominal AT, an elevated expression of miR-24, miR-30d, and miR-146a was reported in subjects with obesity and T2DM and being positively correlated with secreted frizzled-related protein 4 (SFRP-4), an adipocytokine, which could contribute to T2DM by influencing insulin sensitivity and promoting inflammation [344].

Generally, people suffering from obesity display lower oxygen consumption proper to the lower blood flow in their WAT, which turns out to affect macrophages and preadipocytes, subsequently leading to, e.g., dysregulated adipocytokine production, inflammation, WAT fibrosis [345]. Runtsch et al. revealed in recent research that miR-146a protects mice from diet-induced MetS, suggesting its influence on PI3K/AKT/mTOR signaling pathway, glycolysis ROS production and anti-inflammatory effect. miR-146a-/- HFD-fed mice had disrupted blood glucose levels, enhanced weight gain and developed hepatosteatosis [346]. Moreover, miR-146 was proved to target tumor necrosis factor receptor associated factor 6 (TRAF-6), which was suggested to be an signaling axis for regulation of AT macrophages (ATM) inflammation [346]. Accordingly, miR-146a was also indicated to participate in immune response due to suppression of TLR4/NF-κB signaling, and was suggested to serve as an anti-inflammatory protection against diabetic nephropathy [270,347]. Furthermore, Qu et al. have proved that miR-146a inhibits OxS in intracerebral hemorrhage (ICH) rats, suggesting its direct action on the TRAF6/NF-κB pathway. Overexpression of miR-146a also stimulated the activity of SOD and GPx [348]. Visceral obesity-related hypoxia contributed to elevation of miR-128, a direct and proved negative regulator of INSR, and downregulation of INSR levels in obese VAT, leading to repression of glucose uptake and changes in levels of some proinflammatory factors, suggesting its role in inducing systemic IR [349]. Interestingly, in vitro studies showed amelioration of expression changes of INSR and other insulin signaling components upon reoxygenation, thus indicating the reversible nature of hypoxia-induced molecular events [349].

As mentioned earlier, circulating miR-122 is a molecule predicting the risk of developing T2DM and MetS according to a prospective population-based Bruneck study [30]. It is also one of the first miRNAs described in humans due to its abundance in the liver—it rules the miRNA content of the liver, representing 72% of cloned sequences [350]. MiR-122 regulates cationic amino acid transporter 1 (CAT-1), aldolase A (AldoA), N myc downstream regulated gene 3 (Ndrg3), as well as systemic iron homeostasis by repressing the target mRNAs hemochromatosis (Hfe) and hemojuvelin (Hjv) [351,352,353,354]. Furthermore, human Dgat1 (diacylglycerol O-acyltransferase 1) and Agpat1 (1-acyl-sn-glycerol-3-phosphate acyltransferase alpha) mRNAs, known to be involved in triglyceride (TG) synthesis, are targets of miR-122. Chai et al. suggested that FFAs elevated miR-122 levels in livers of mice by inducing activation of retinoic acid-related orphan receptor alpha (RORα), then generating miR-122 secretion from the liver to blood [355]. Further, upregulation of miR-122 lessened TG biosynthesis and increased β-oxidation, shifting lipid accumulation to an energy source and pointing out miR-122 as a therapeutic target in MetS [355]. In contrast, Elmen et al. reported that anti-miR-122 therapy in mice and non-human primates led to considerably lower plasma cholesterol levels. This could be linked with expression changes of genes associated with cholesterol synthesis such as squalene epoxidase (SQLE), HMGCR, 3-hydroxy-3-methylglutaryl-CoA synthase 1 (HMGCS1), and 7-dehydrocholesterol reductase (DHCR7), although these genes are not direct target of miR-122 [356]. It was also reported that the lack of miR-122 underlies reduced expression of MTP in this way leading to limited secretion of VLDL from liver [357]. In this study, miR-122 was confirmed to target Krüppel like factor 6 (KLF6), a gene associated with fibrosis in NAFLD. As proved in rat model of liver injury, mir-122 is associated with OxS [358].

Another important miRNA in lipid metabolism is miR-132. Transgenic mice model with overexpression of miR-132 was characterized by severe fatty liver phenotype along with increased body weight, triglycerides, serum low-density lipoprotein/very low-density lipoprotein (LDL/VLDL) and insulin [359]. It is also upregulated in livers of NAFLD and mice models with NASH and hepatic steatosis while targeting SIRT1, PTEN, histone acetyltransferase P300 (P300), FOXO3, cytochrome P450 family 2 subfamily E member (CYP2E1) [359]. For instance, PTEN, SIRT, FOXO3 were linked with hepatic steatosis, hyperlipidemia, and glucose regulation [360,361,362]. Several studies reported association between OxS and miR-132 regulation [362,363,364].

Interestingly, Meiler and collaborators reported that miR-302a is a regulator of atherosclerosis and cholesterol homeostasis, being inversely correlated with ABCA1 and ABCG1 in primary macrophages and PBMCs [365]. Moreover, miR-302a was subjected to significant downregulation upon acetylated (ac)-LDL and ox-LDL stimulation. Researchers validated the experimentally targeting of ABCA1 by miR-302a. In the experiment involving usage of anti-miR-302a both ABCA1 protein expression and efflux of cholesterol to apoA-1 were elevated in murine and human macrophages. This type of treatment elicited raise in HDL levels and moderated progression of atherosclerosis in Ldlr−/− mice. Moreover, several studies have shown that decline of miR-302 has a positive impact on OxS reduction. Fang et al. observed that miR-302 expression was elevated by hypoxia/reoxygenation injury and mediated cardiomyocytes apoptosis by inhibiting antiapoptotic myeloid cell leukemia 1 (MCL-1) expression and activating proapoptotic molecules [366].

Lipid regulating hormone, angiopoietin-like 3 (ANGPTL3), a known regulator of circulating apolipoproteins through inhibition of the lipoprotein lipase activity, is involved in adipocyte differentiation [367]. ANGPTL3 inhibitors are perceived as potential therapeutics to reduce plasma lipid levels. Abu-Farha et al. have shown reduction of miR-181d levels in obese in comparison to non-obese individuals. In vitro analysis confirmed miR-181 binding and repression of the ANGPTL3 transcript [368]. Wang et al. have shown the upregulation of miR-181a, a miRNA involved in direct targeting of GPx-1, in the H9c2 rat cardiomyocyte cell line exposed to H2O2. Downregulation of miR-181a prevented H2O2-promoted apoptosis, production of ROS and MDA, rupture of mitochondria, as well as activation of signaling molecules involved in mitochondrial apoptotic pathway [22].

**Figure 5:**
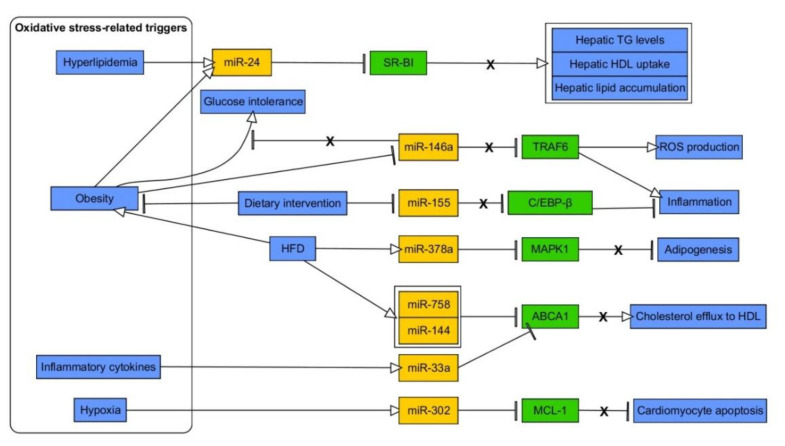
Proposed model of phenomena linking oxidative stress and obesity-related miRNAs. Stimulatory interactions are indicated by arrows and inhibition by T-bars. Actions related to boxes refer to all items inside the box. Arrows and T-bars which are crossed out denote the withdrawal of the respective type of regulation. Processes/phenomena are highlighted in blue, proteins/compounds are highlighted in green and miRNAs are highlighted in yellow. The figure was made using the PathVisio 3.3.0 free open-source software [64]. miR—microRNA, SR-BI—scavenger receptor class B type I, HFD—high-fat diet, TRAF6—tumor necrosis factor receptor (TNFR)-associated factor 6, C/EBP-β—CCAAT/enhancer-binding protein β, MAPK1—mitogen-activated kinase 1, ABCA1—ATP-binding membrane cassette transporter A1, MCL-1 —myeloid cell leukemia 1, TG—triglycerides, HDL—high-density lipoprotein.

Results obtained by Shen et al. suggested that miR-144-3p may be a potential target for therapeutic intervention in obesity and MetS. They indicated that the overexpression of miR-144-3p stimulated adipocyte lipid accumulation and exerted a positive impact on adipogenesis, being accompanied not only by an upregulation of genes involved in the synthesis of fatty acids, but also the downregulation of genes related to fatty acid oxidation. For instance, genes linked with fatty acid synthesis targeted by miR-144-3p are KLF3 and carboxy-terminal binding protein 2 (CtBP2). Overexpression of miR-144-3p could significantly increase serum levels of total TG, TC, and LDL when compared to the healthy probands [369]. Interestingly, in conditions of Alzheimer’s disease-associated OxS, miR-144 reduced cellular viability, enhanced ROS accumulation, exerted negative impact on activity of GSH and GPx-1 along with diminishing expression of Nrf2, GCLM, and GCLC [370]. miRNAs described in this section are briefly summarized in Table 3, and some of them were included in Figure 5.

### 3.4. MicroRNA in MetS—A Link with Endothelial Dysfunction and Hypertension Related to Oxidative Stress

Endothelial dysfunction, characterized by imbalance between production of vasodilators and vasoconstrictors and reduced production of NO, plays a pivotal role in the pathophysiology of arterial HT, contributes to the development of atherosclerosis, and is regulated by numerous miRNAs [371,372]. One of the miRNAs connected with OxS and endothelial dysfunction is miR-1. Its upregulation in pulmonary arteries induced ROS and led to direct targeting of SOD1, and downregulation of its other predicted target genes such as connexin-43 (Cx43), KLF4 and caveolin 2 (CAV2) mRNA leading to endothelial dysfunction [373]. Mondejar-Parreño et al. showed that miR-1 upregulation reduces the activity and expression of voltage-dependent potassium channel (Kv)1.5. (encoded by KCNA5) while inducing hypertrophy of pulmonary artery smooth muscle cells, thus suggesting a pathophysiological role in pulmonary arterial hypertension (PAH) [374]. Also, other studies have shown that miR-1 aggravates antioxidative response [375,376,377]. For instance, according to Wang and collaborators, the overexpression of miR-1 leads to mice heart dysfunction related to decreased protein and mRNA expression of 3 redox-related molecules (GCLC, SOD1, and G6PD) under OxS [375]. miR-1 was also reported to directly target myosin light chain kinase (MLCK) in HUVECs in the study showing ox-LDL-induced miR-1 upregulation and MLCK downregulation in a time- and dose-dependent manner [378]. Interestingly, ox-LDL was shown to increase expression of miR-34a or miR-106a-5p, and decrease levels of direct regulators of TLR4 (miR-20a, miR-221-3p) along with deterioration of OxS in affected endothelium [379,380,381,382].

Dluzen et al. revealed that miRNAs linked to HT, miR-103a-2-5p and miR-585-5p, downregulate poly-(ADP-ribose) polymerase 1 (PARP-1) [383]. The authors have previously found that PARP-1 protein is upregulated in the peripheral blood mononuclear cells (PBMCs) of African American women with HT [384]. PARP-1′s primary role is to detect DNA damage and initiate DNA repair processes. Consistently, overexpression of mentioned miRNAs resulted in increased DNA damage and reduced viability of endothelial cells [383]. Interestingly, knockdown of proved NOX4-targeting miRNAs (miR-92a-3p, miR-92b-3p, miR-99-5p, and miR-100-5p) enhanced H2O2 release from HMVEC-D (hypertension in endothelial cell line) cells. Moreover, knockdown of miR-21 by intraperitoneal administration of anti-miR-21 resulted in reduced murine blood pressure [385]. Oppositely, in another study, administration of miR-21 mimic lowered blood pressure in spontaneous hypertensive Wistar rats by targeting and upregulating cytochrome b (Cytb) in heart mitochondria. Authors suggested that elevated levels of miR-21 in patients with HT is an element of the compensatory mechanism. Furthermore, transfection of miR-21 to H9c2 cells reduced mitochondrial ROS production [386]. On the other hand, elevated levels of miR-21 in hypertensive patients were observed in the next study, whereas eNOS levels were decreased as compared to healthy controls. miR-21 was detected to be positively correlated with blood pressure and carotid intima media thickness, however, eNOS levels were negatively correlated with miR-21 [387]. Another miRNA, miR-155, was revealed to participate in inflammation-induced impairment of endothelial-dependent vasorelaxation, and hence putative development of cardiovascular diseases. Moreover, miR-155 was proved to downregulate expression of eNOS using HUVECs [388]. Interestingly, miR-140-5p induced OxS, by targeting Nrf2 and SIRT2, and exacerbated HT in mice with atherosclerosis [389]. miRNAs described in this section are briefly summarized in Table 4.

## 4. Concluding Remarks and Future Perspectives

**Figure 6:**
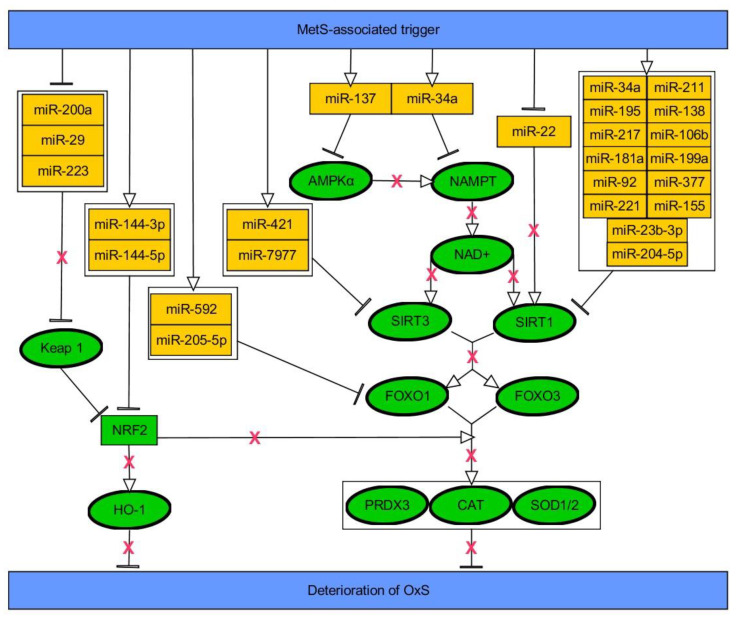
The impact of MetS-associated triggers on miRNAs involved in regulation of major antioxidant signaling pathways. Stimulatory interactions are indicated by arrows and inhibition by T-bars. Actions related to boxes refer to all items inside the box. Arrows and T-bars which are crossed out denote the withdrawal of the respective type of regulation. Processes/phenomena are highlighted in blue, proteins/compounds are highlighted in green and miRNAs are highlighted in yellow. The figure was made using the PathVisio 3.3.0 free open-source software [64]. miR–microRNA, AMPKα–5’AMP-activated protein kinase, NAMPT—nicotinamide phosphoribosyltransferase, SIRT1/3–sirtuin 1/3, FOXO1/3—forkhead box O1/3, CAT—catalase, SOD1/2—superoxide dehydrogenase 1/2, PRDX3—peroxiredoxin3, HO-1—heme oxygenase 1, Keap1—kelch-like ECH-associated protein 1, NRF2—nuclear factor erythroid 2-related factor 2.

**Figure 7:**
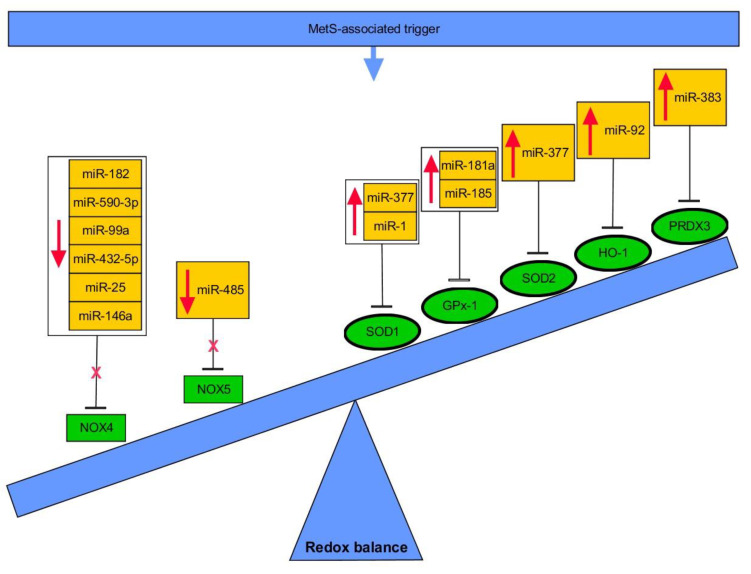
The miRNA-mediated redox imbalance under MetS-associated triggers. Stimulatory interactions are indicated by arrows and inhibition by T-bars. Actions related to boxes refer to all items inside the box. T-bars which are crossed out denote the withdrawal of the inhibition. Red arrows denote either upregulation or downregulation of expression levels. Processes/phenomena are highlighted in blue, proteins/compounds are highlighted in green and miRNAs are highlighted in yellow. The figure was made using the PathVisio 3.3.0 free open-source software [64]. miR–microRNA, NOX4/5—NADPH oxidase 4/5, CAT—catalase, SOD1/2—superoxide dehydrogenase 1/2, PRDX3—peroxiredoxin 3, HO-1—heme oxygenase 1.

In the course of MetS, obesity, hypoxia, HG, IR, dyslipidemia, HT, and proinflammatory cytokines induce OxS that can no longer be modulated by cellular antioxidative response, thus leading to the excessive generation of ROS and cellular damage. Collected data strongly indicates that OxS, generated in MetS or upon its components, changes expression level of numerous miRNAs in endothelium and tissues critical for glucose and lipid metabolism as well as IR formation. MetS-associated triggers were shown to change expression of miRNAs targeting the three most important molecular cytoprotective modules, namely Nrf2, SIRT1, and FOXOs (Figure 6). Furthermore, due to the fact that miRNAs regulate numerous signaling pathways associated with glucose (e.g., PTEN) and lipid (e.g., PPARγ) metabolism, inflammation (e.g., NF-κB, JNK), as well as directly modulate the expression of ROS-scavenging (e.g., SOD1/2, GPx-1, HO-1) and ROS-generating enzymes (NOX4/5), they constitute important molecules in balancing the cellular redox state in metabolic disorders (Figure 7). While there is a plethora of miRNAs found to regulate NOX4, the data regarding relation between miRNAs and other prooxidant enzymes is still scarce. Therefore, there is a great need to perform immense studies on the interplay between OxS and miRNAs, as there remain questions about their role as antioxidative agents. Interestingly, miRNAs regulating OxS are more and more common to be regulated by lncRNAs, showing the bigger picture of phenomena associated with non-coding RNAs and their biological relevance. Specifically, the expression of LEGLTBC, HOTAIR, lncRNA H19, GAS5, MEG3, SNHG7, and LINC01619 was reported to be affected by diabetic milieu, thus changing their capabilities of serving as sponges for miR-34a, miR-29b, miR-452-5p, miR-221, miR-93, miR-543, and mir-27a. Due to the limited amount of studies, the relation between lncRNAs and miRNAs associated with MetS and OxS appears to be especially attractive for researchers studying other triggers of OxS (e.g., hyperlipidemia).

Taken together, in the context of profound interest in the search for personalized, miRNA-based therapeutics and the increasing possibilities of using miRNAs as biomarkers for the evaluation of disease progression and effectiveness of therapy, we are truly convinced that miRNAs related to OxS constitute a group of particularly promising molecules in MetS.

## Figures and Tables

**Table 1:** Summary of miRNAs and their validated targets changed upon and/or associated with diabetic milieu and/or obesity from Section 3.1.

miRNA	Up/Down Regulation of Proved Target (UP/DOWN) upon OxS	Validated Target	Changed Expression and/or Activity of Non-Direct Targets	Human (H)/Rodent (R)/In Vitro/In Vivo Study Model	Ref.
miR-375	#	MTPN, Pdk1	-	R, in vivo, in vitro	[153]
miR-9	#	STXBP1	-	R, in vivo, in vitro	[156]
miR-29a/c	#	-	-	R, in vivo	[155]
miR-15	##/UP	AKT3	-	H, in vivo, and R, in vivo, in vitro	[158]
miR-377	UP	SIRT1	Decreased AKT and ERK phosphorylation and increased levels of proinflammatory factors	R, in vivo, in vitro	[172]
miR-34a ^1^	UP	SIRT1	Decreased signaling via SIRT1/FOXO1 pathway	R, in vivo, in vitro	[178]
miR-34a ^2^	UP	SIRT1	-	R, in vivo, in vitro	[179]
miR-34a ^3^	UP	SIRT1	-	H, in vitro	[180]
miR-34a	UP	SIRT1, Bcl2	-	R, in vitro	[181]
miR-195	UP	SIRT1	-	H, in vitro and R, in vivo	[281]
miR-217	UP	SIRT1	Simultaneous increase of HIF1α	R, in vitro	[282]
miR-155	UP	SIRT1	-	R, in vivo	[283]
miR-204-5p	UP	SIRT1	Simultaneous reduction of cyclin D1 and increase of p16	R, in vivo, in vitro	[284]
miR-211	UP	SIRT1	Simultaneous Bcl-2 and Bax decline and increase of p53 Bax	R, in vivo	[285]
miR-23b-3p	UP	SIRT1	Simultaneous downregulation of Nrf2	R, in vivo, in vitro	[182]
miR-221 ^4^	UP	SIRT1	Simultaneous downregulation of Nrf2	H, in vitro	[183]
miR-221	UP	SIRT1	miR-221 inhibition elicited reduction of fibronectin, collagen 4 and TGFβ1	R, in vivo, in vitro	[184]
miR-181a	UP	SIRT1	Overexpressed miR-543, miR-30a, miR-199b and miR-200a also decreased the activity of 3′UTR of SIRT1 (luciferase assay), yet the impact of miR-181a was the most pronounced	H, R, in vivo, in vitro	[186]
miR-182	DOWN	NOX4	SIRT1 was proved to be positive miR-182 regulator	R, in vitro, in vivo	[187]
miR-138	UP	SIRT1	Decreased signaling via PI3K/AKT and AMPK pathways	H, in vitro	[188]
miR-543 ^5^	UP	SIRT1	Simultaneous decrease of VEGF	H, in vitro	[189]
mir-106b-5p	UP	SIRT1	Simultaneous decrease of SOD1 protein in islets of diabetic mice	R, in vitro, in vivo	[190]
miR-199a-5p	UP	SIRT1	-	R, in vitro	[191]
miR-22 *	DOWN	SIRT1	-	R, in vitro, in vivo	[185]
miR-7977	UP	SIRT3	-	H, in vitro, in vivo	[194]
miR-27a	UP	PPAR-γ	Downregulation of PPAR-γ / PI3K / AKT / GLUT4 signaling.	R, in vitro, in vivo	[200]
miR-592	DOWN	FOXO-1	-	H, R, in vitro, in vivo	[201]
miR-708-5p	UP	NNAT	-	R, in vitro, in vivo	[203]
miR-483-5p (co-expressed with IGF2)	-	SOCS3	-	R, in vitro, in vivo	[204]
miR-194	DOWN	-	-	H, in vivo	[210]
miR-192, miR-194, miR-215	UP	-	-	H, in vivo	[213]
miR-802	UP	-	Declined activity of SOD, CAT, GPx, and increase of phosphorylated p38MAPK and JNK	R, in vivo	[214].
miR-233	DOWN	Keap1	Reduction of Nrf2, HO-1 and SOD1	H, in vitro,	[215]
miR-99a	UP	NOX4	-	R, in vivo	[216]
miR-21	UP	Bcl-2	-	R, in vitro, in vivo	[221]
miR-21	UP	-	Decrease of SOD2, Nrf2 and KRIT1	H, in vitro	[225]
mir-21	UP	-	Reduced antioxidant activity of SOD2	H, in vivo	[224]
miR-200c	UP	ZEB1	-	R, in vivo	[231]
miR-185	UP	GPx-1	-	H, in vitro	[242]
miR-155	UP	eNOS	Increased NF-κB signaling and repressed signaling via Nrf2/HO-1	H, in vitro	[246]
miR-29b ^6^	DOWN	VEGFA	Associated with decreased signaling via AKT/eNOS pathway	H, in vitro, in vivo	[245]
mir-92a	UP	HO-1	-	H, in vitro and R, in vivo, in vitro	[250]
miR-200a/b	DOWN	OGT	-	H, in vitro and R, in vivo	[247]
miR-200a	DOWN	Keap1	Decreased signaling of Nrf2	R, in vitro, in vivo	[248]
miR-137	UP	AMPKα1	-	H, in vitro	[249]
miR-24	DOWN	OGT	Upregulation of Keap1 and downregulation of Nrf2 and HO-1	R, in vitro, in vivo	[251]
miR-106a	DOWN	12/15-LOX	-	R, in vitro, in vivo	[252]
miR-590-3p	DOWN	NLRP1, NOX4	-	H, in vitro, in vivo	[253]
miR-145	DOWN	TLR4	Increased signaling via TLR4/NF-κB pathway	H, in vitro	[254]
miR-455-5p	DOWN	SOCS3	-	H, in vitro	[255]
miR-29b	DOWN	-	Decreased signaling via PTEN/AKT and increased signaling via NF-κB	H, in vitro	[256]
miR-383	UP	PRDX3	-	H, in vitro	[257]
miR-144-3p/-5p	UP	Nrf2	Declined levels of GR, GCLC, and NQO1	H, in vitro and R, in vivo	[258]
miR-93 ^3^	UP	Nrf2	-	H, in vivo, in vitro	[259]
miR-26a	DOWN	-	Enhanced signaling via ERK and Wnt/β-catenin pathways	H, in vitro	[260]
miR-195	UP	Bcl-2	-	H, R, in vitro	[261]
miR-195	UP	MFN2	-	H, in vitro and R, in vivo	[262]
miR-130a-3p miR-301a-3p	DOWN	TNF-α	-	R, in vitro	[264]
miR-15b-5p	DOWN	Sema3A	-	R, in vitro	[265]
miR-423-5p	DOWN	NOX4	Increased signaling of p38 MAPK	H, in vivo and R, in vitro	[266]
miR-25	DOWN	NOX4	Upregulation of AGE/RAGE axis and PKC-α signaling	R, in vitro, in vivo	[267,268]
miR-485	DOWN	NOX5	Increased expression of proinflammatory cytokines (TNF-α, IL-6, and IL-1β), ECM proteins (collagen IV and fibronectin) and declined activity of SOD	H, in vitro	[269]
miR-146a	DOWN	NOX4	Overexpression of mir-146a elicited decrease of ICAM-1 and VCAM-1	H, in vitro and R, in vivo	[271]
miR-214	DOWN	UCP2 *	ROS-mediated declined signaling via Akt/mTOR	H, in vitro and R, in vivo	[274]
miR-140-5p	DOWN	TLR4	Increased signaling via TLR4/NF-κB	H, in vivo, in vitro	[275]
miR-125b	UP	ACE2	Induction of Bax and inhibition of Bcl-2	H, in vitro	[276]
miR-452-5p ^4^	UP	-	-	H, in vitro	[277]
miR-27a ^7^	UP	FOXO1	-	H, in vivo and R, in vivo, in vitro	[278]
miR-203	DOWN	PI3KCA	-	R, in vivo, in vitro	[279]
miR-92a-2-5, let-7b-5p	DOWN	mt-Cytb * IRS1 ###	-	R, in vivo, in vitro	[280]

(#) miRNAs involved in insulin secretion, (##) miRNA involved in insulin production, (^1^) miRNA regulated by lnRNA (HOTAIR), (^2^) miRNA regulated by lnRNA (LEGLTBC) (*) miRNAs-induced positive regulation of expression, (^3^) miRNA regulated by lnRNA (MEG3), (^4^) miRNA regulated by lnRNA (lncRNA GAS5), (^5^) miRNA regulated by lnRNA (SNHG7), (^6^) miRNA regulated by lnRNA (H19), (^7^) miRNA regulated by lnRNA (LINC01619), (###) regulated only by let-7b-5p.

**Table 2:** Summary of miRNAs and their validated targets changed in MetS with and without dietary interventions, NAFLD or associated with chronic inflammation and described in Section 3.2.

miRNA	Up/Down Regulation	Validated Target	Summary	Human (H)/Rodent (R)/In Vitro/In Vivo Study Model	Ref.
miR-223, miR-146a	DOWN	-	Changes observed in PBMCs of MetS patients vs control subjects	H, in vivo	[286]
	DOWN				
miR-21	UP	-			
miR-155-3p	DOWN	-	Changes evoked in WBCs of MetS patients after 8 weeks of Mediterranean diet	H, in vivo	[289]
let-7b	UP	-			
miR-155-5p miR-34a-5p	UP	-	Changes reported in adipocytes exposed to TNF-α and their exosomes, being prevented by pretreatment with Mediterranean-diet phytochemical (hydroxytyrosol)	H, in vitro	[293]
	UP				
let-7c-5p	DOWN				
	
miR-424	UP	-	Observed in serum of patients with T2DM after intervention with fruit juice of *Actinidia chinensis* planch (kiwi) and correlated positively with levels of SOD and GSH	H, in vivo	[294]
miR-377	UP	SOD1, SOD2	Changes observed in MetS-associated podocyte injury upon exposure to high fructose	R, in vitro, in vivo	[300]
miR-10a	DOWN	LCoR, Ncor2	-mediates the impact of DICER on adaptation of macrophages to an excess of fatty acids via being involved in mitochondrial fatty acid oxidation	H, R, in vivo	[303]
			-declined in human carotid plaques (vs vessel walls) exerts atheropreotective role		
miR-421	UP	SIRT3	Observed in NAFLD and triggering disturbed signaling of FOXO1 and decrease of SOD2 and CAT	R, in vivo, in vitro	[306]
miR-34a	UP	SIRT1	Downregulation of SIRT1 elicits repression of fatty acid oxidation and deterioration of hepatic lipid accumulation via affecting SREBP, MLYCD, and CPT1	R, in vivo	[307,309]
				H, in vitro, in vivo, and R, in vivo	
miR-34a	UP	SIRT1	Changes observed upon high-fructose diet leading to upregulation of SREBP protein and mRNA levels of FAS, SCD1, and thus, hepatic lipid accumulation. This outcome was ameliorated by pterostilbene	H, in vitro and R, in vitro, in vivo	[312]
miR-34a	UP	NAMPT	Upregulated hepatic miR-34a triggers decline of SIRT1 activity. Antagonism of this miRNA ameliorated glucose tolerance, inflammation and steatosis in obese mice	R, in vivo, in vitro	[297]
miR-23b-3p	-	SIRT1	miR-23b-3p downregulates SIRT1 to increase hepatic lipid accumulation	H, in vitro	[310]
miR-9-3p	-	-	Overexpressed miR-9-3p downregulates only protein levels of SIRT1 to increase hepatic lipid accumulation	H, in vitro	[311]
miR-200a	DOWN	Keap1	Elicited upon high-fructose diet, and leading to reduction of Nrf2 signaling and downregulation of HO-1, GST and NQO1	R, in vivo, in vitro and H, in vitro	[313]
miR-29a	DOWN	CD36	Increased CD36 leads to potentiated lipid flux into the liver and PPARγ-mediated increase of mtDNA and mitochondrial ROS	R, in vivo	[314]
miR-21a-5p	DOWN	-	Changed with other miRNAs (miR-101b-3p, miR-455-5p, and increased let-7a-5p,) upon EPA + HFD treatment in hepatocytes, being suggested to participate in improvement of hepatic metabolism and inflammation.	H, in vitro, and R, in vivo	[316]
miR-101	UP	ABCA1	Observed upon IL-6 and TNF-α treatment, supporting intracellular cholesterol retention	H, in vitro	[317]
miR-9-5p	UP	ABCA1	-a NF-κB target upregulated in MetS patients’ CD14+ cells,	H, in vivo	[322]
			-stays in positive correlation with BMI, TG and HOMA-IR, and may serve as a potent anti-atherosclerotic player in MetS		
miR-128-2	DOWN	ABCA1, ABCG1, RXRα, SIRT1	-declined miR-128-2 in HFD-fed mice	R, in vivo	[125]
			-miR-128-2 leads to upregulation of SREBP-2, but reduction of SREBP-1		
			-overexpression of miR-128-2 reduces cholesterol efflux		
miR-33a-5p	UP	ABCA1, ABCG1	Observed upon IL-6 and TNF-α treatment with and without presence of LDL in macrophages, supporting cholesterol efflux and lipid accumulation	H, in vitro	[323]
miR-146b	UP	-	IL-6 and TNF-α activate promoter regions of miR-146b in visceral adipocytes	H, in vitro	[324]
miR-130a/b	UP	PPAR-γ	Increased upon TNF-α treatment in adipocytes via binding of p68 subunit of NF-κB. Increased in AT of HFD-mice	R, in vitro, in vivo	[325]
miR-155	UP	PPAR-γ	-Induced by TNF-α in adipocytes in a NF-κB-dependent way (p68 subunit) and in AT of the obese subjects	H, R, in vitro, in vivo	[326]
			-responsible for induction of chemokine expression, inflammatory response, and macrophage migration in mice adipocytes		
miR-199a-3p	UP	-	-Increased in visceral AT of obese probands and visceral adipocytes upon exposure of FFA, TNF-α, IL-6, leptin, but decreased with resistin	H, in vivo, in vitro	[327]

**Table 3:** Summary of miRNAs and their validated targets changed by MetS, obesity, hyperlipidemia and hypercholesterolemia, involved in lipid metabolism and described in Section 3.3.

miRNA	Up/Down Regulation	Validated Target	Summary	Human (H)/Rodent (R)/In Vitro/In Vivo Study Model	Ref.
miR-25	DOWN	NOX4	Observed upon hypercholesterolemia in rat hearts leading to diastolic dysfunction and OxS/NS	R, in vivo R, in vivo, in vitro	[21]
47 miRNAs	UP	-	In hypercholesterolemic hearts microarray analysis reported upregulated miRNAs (e.g., miR-133b, miR-101a, miR-29b, miR-223, miR-21) and downregulated miRNAs (e.g., miR-93, miR-25)	R, in vivo	[329]
and					
10 miRNAs	DOWN	-			
miR-125b-1-3	-	-	Hypercholesterolemia prevented increase of the miRNA after ischemic preconditioning	R, in vivo	[328]
miR-98	DOWN	SREBP-2	Observed in hypercholesterolemic patients (serum and liver). miR-98 overexpression elicited decline of SREBP, LDLR, and HMGCR in mice	H, R, in vivo	[330]
miR-92	UP	SIRT1, KLF2, KLF4	-H2O2, Ang II, and ox-LDL increased miR-92 and SREBP-2 in HUVECs, promoted targeting of SIRT1, KLF2/4 changing NOS-NO bioavailability and endothelial innate immunity	H, R, zebrafish, in vivo	[331]
			-High cholesterol diet elicited SREBP-2-dependent increase of miR-92		
miR-379	UP	-	Serum level positively correlated with high cholesterol, predicted to target numerous genes critical for metabolism	H, in vivo	[332]
miR-27a	UP	HMGCR	-Hypoxia induces Egr-1/miR-27a axis, leading to downregulation of HMGCR.	R, in vivo	[334]
			–upregulated also in livers of 3 mice models of MetS		
			-HMGCR targeting was proved in various mammalian species-derived cell lines		
miR-30c	-	-	In livers of *Apoe*^−/−^ mice fed a Western diet, miR-30c mimic triggered decrease of cholesterol levels and putative target genes (Elovl5, Mttp, QKI, LPGAT1)	R, in vivo	[335]
	-	LPGAT1, MTP	These expression changes elicit induction of hepatic lipid synthesis and apoB secretion. It may serve as an anti-hyperlipidemic as well as anti-atherosclerotic molecule	R, in vivo	[336]
miR-155-5p	UP	Mafb	Increased by hyperlipidemia to adapt β-cells to IR. Triggered reduction of IL-6 and consequent inhibition of intra-islet production of GLP-1	H, in vitro and R, in vivo, in vitro	[337]
miR-24	UP	SR-BI	-Increased in livers under obesity and hepatocytes under hyperlipidemic conditions	H, in vitro and R, in vivo	[338]
			–deteriorates HDL uptake and affects lipid metabolism		
miR-125a miR-455	-	SR-BI	miRNAs involved in negative regulation of HDL cholesteryl ester (HDL-CE) uptake	R, in vivo, in vitro	[342]
miR-125a	DOWN	Elovl6	Decreased by obesity in liver, yet, if overexpressed ameliorates hepatic steatosis, lipid accumulation and increases insulin sensitivity	R, in vivo	[343]
miR-24 miR-30d miR-146a	UP	-	Increased in abdominal AT in obese and T2DM subjects, potentially coregulated due to strong positive correlation among them. Positively correlated with SFRP-4	H, in vivo	[344]
miR-146a	-	TRAF-6	-miR-146 knockout mice were protected from MetS upon HFD via influencing PI3K/AKT/mTOR axis.	R, in vivo	[346]
			–by targeting TRAF-6, miR-146a regulates ATM inflammation		
miR-128	UP	INSR	Increase elicited upon VAT hypoxia and suggested to participate in induction of systemic IR	H, R, in vivo, in vitro	[349]
miR-122	UP	Agpat1, Dgat1	FFA increase miR-122 in mice liver via RORα-dependent way. miR-122 is then secreted to increase AT and muscle TG synthesis by targeting Agpat1 and Dgat1	R, in vivo	[355]
	-	-	Therapy with anti-miR-122 results in lower levels of cholesterol	R, in vivo	[356]
	-	KLF3	miR-122 knockout mice showed declined expression of MTTP, leading to disturbance of lipid profile (e.g., VLDL secretion). KLF3 is a another gene critical for liver homeostasis and associated with miR-122	R, in vivo	[357]
miR-132	UP	SIRT1, PTEN, P300, FOXO3, CYP2E1	Regarded as key player in hepatic lipid homeostasis, may serve as human and mice biomarker of NAFLD and NASH. Its overexpression was accompanied by decline of its earlier validated targets	H, R, in vivo	[359]
miR-302	DOWN	ABCA1	Reduced by ac-LDL and ox-LDL, mediating increased cholesterol efflux to macrophages	H, in vivo and R, in vivo	[365]
	UP	MCL-1	Increased by hypoxia/reoxygenation injury, triggering apoptosis of cardiomyocytes	R, in vitro	[366]
miR-181d	DOWN	ANGPTL3	Downregulated in serum and AT of obese subjects and negatively correlated with TG. Increased ANGPTL3 represses lipolysis via LPL	H, in vivo	[368]
miR-181a	UP	GPx-1	Increased by H2O2 in cardiomyocytes	R, in vitro	[22]
miR-144-3p	UP	KLF3, CtBP2	Increased in AT of obese mice, positively impacts adipogenesis (releases C/EBPα from KLF3, CtBP2) and fatty acid synthesis and decreases genes of FAO	R, in vivo, in vitro	[369]

**Table 4:** Summary of miRNAs linked to endothelial dysfunction, hypertension and described in Section 3.4.

miRNA	Up/Down Regulation	Validated Target	Summary	Human (H)/Rodent (R)/In Vitro/In Vivo Study Model	Ref.
miR-1	UP	SOD1	Decline of SOD1, Cx43, KLF4 and CAV2 in models of pulmonary HT	R, in vivo, in vitro	[133]
miR-1	UP	Kv1.5 channels	Reduction of expression and activity of Kv1.5 channels in pulmonary artery smooth muscle cells was accompanied by membrane depolarization	R, in vivo, in vitro	[134]
miR-1	UP	-	Decrease of expression of GCLC, SOD1, and G6PD under OxS evoked by myocardial ischemia	R, in vivo	[135]
miR-1	UP	MLCK	Reduction of MLCK and phosphorylation of MLC and ERK/p38 MAPK upon ox-LDL	H, in vitro	[136]
miR-34a	UP	Bcl-2	Increased in atherosclerotic plaques and serum and upon exposure to ox-LDL in HUVECs. miR’s knockdown protected from apoptosis and ameliorated ROS production	H, in vivo, in vitro	[120]
miR-106a-5p	UP	STAT3	Reported ox-LDL-evoked expression changes were accompanied by increased ROS accumulation and apoptosis of endothelial cells	H, in vitro	[121]
miR-20a	DOWN	TLR4	Overexpression of miR-20a elicited decline of numerous inflammation-related genes in endothelial cells exposed to ox-LDL	H, in vitro	[125]
miR-221-3p	DOWN	TLR4	Observed expression changes were associated with inflammation, apoptosis and OxS in HUVECs treated with ox-LDL	H, in vitro	[124]
miR-103a-2-5p	-	PARP	Expression of the miRNAs was unchanged in PBMCs of hypertensive women, yet their overexpression showed increased DNA damage	H, in vivo	[126]
miR-585-5p					
miR-21	-	-	-Treatment with anti-miR21 elicited reduction of blood pressure in mice treated with a 4% NaCl diet.	R, in vivo	[128]
miR-21	UP	-	Increased circulating level of miR-21 in hypertensive subjects	H, in vivo	[129]
	-	Cytb	-miR-21 mimic elicited Cytb and blood pressure reduction in spontaneous rat model of HT	R, in vivo	
miR-21	UP	-	Increased serum level of miR-21 in hypertensive patients vs control subjects. miR-21 level was negatively correlated with NOx and eNOS levels	H, in vivo	[130]
miR-155	UP	eNOS	-Increased upon TNF-α in HUVECs	H, in vitro	[131]
			-Triggered vasorelaxation		
			-Downegulated by simvastatin		
miR-140-5p	UP	Nrf2, SIRT2	-Observed expression changes were connected with augmentation of HT in mice suffering from atherosclerosis –Overexpression of miR-140-5p also elicits downregulation of non-target proteins such as Keap-1 and HO-1	R, in vivo	[132]
